# Supplementary material for: Empirical methods for controlling false positives and estimating confidence in ChIP-Seq peaks
Source: BMC Bioinformatics. 2008 Dec 5;9:523. doi: 10.1186/1471-2105-9-523 (PMC2628906; doi:10.1186/1471-2105-9-523)
Supplement: Additional File 1 — A variety of html documents from the USeq web site detailing the available applications, their best usage, output file type descriptions, command line menus, etc. [file 1471-2105-9-523-S1.zip › USeqUserGuides/JavaDoc.zip!/JavaDoc/index-files/index-7.html]

 
 
 
 
 
 
G-Index
 


 

 
function windowTitle()
{
    parent.document.title="G-Index";
}
 
 
 

 

 


 
   
  
 
 
 
   
 
   
          Overview     
        Package   
        Class   
        Use   
          Tree     
          Deprecated     
       Index    
          Help     
   
 
 
  
 
 
 

 
  
   PREV LETTER   
   NEXT LETTER    
  
    FRAMES     
   NO FRAMES     
  
    All Classes  ');
  }
  //-->
 
 
    All Classes  
 


  
 
 
  
 

 A   B   C   D   E   F   G   H   I   J   L   M   N   O   P   Q   R   S   T   U   V   W   X   Z   
    
 G  
 
   GadFlyGffExtractor   - Class in  util.bio.parsers.gff  Class for building annotation objects from GffFeatures, basically a converter, specific to the old dmel GadFly annotation.   GadFlyGffExtractor(File, int, int)   - 
Constructor for class util.bio.parsers.gff. GadFlyGffExtractor 
  
   gata.aligner   - package gata.aligner For aligning and post processing DNA sequences using BLAST for subsequent import into GATAPlotter.   gata.geneGlyphs   - package gata.geneGlyphs For representing annotation, scale bars, and generic tracks.   gata.main   - package gata.main Main app, utility, and params for GATAPlotter.   gata.menu   - package gata.menu GATAPlotter menus, panels, and frames.   gata.plotter   - package gata.plotter GATAPlotter primary panel, sliders, tools, and conservation panels.   GATAFrame   - Class in  gata.menu      GATAFrame(GATAParams)   - 
Constructor for class gata.menu. GATAFrame 
  
   GATAligner   - Class in  gata.aligner      GATAligner()   - 
Constructor for class gata.aligner. GATAligner 
  
   GATAParams   - Class in  gata.main      GATAParams()   - 
Constructor for class gata.main. GATAParams 
  
   GATAPlotter   - Class in  gata.main      GATAPlotter()   - 
Constructor for class gata.main. GATAPlotter 
  
   GATAUtil   - Class in  gata.main      GATAUtil()   - 
Constructor for class gata.main. GATAUtil 
  
   gatherInfo()   - 
Method in class trans.tpmap. MapSplitter 
  
   GCRatio   - Class in  trans.misc  Container for a ratio and associated %GC content for a particular oligo.   GCRatio(double, int)   - 
Constructor for class trans.misc. GCRatio 
  
   genDashes(String, String)   - 
Static method in class gata.main. GATAUtil 
  
   genDashes(String, String)   - 
Static method in class util.bio.seq. Seq 
 Generates identitiy dashes (ie"|||  | |||") between two aligned sequences
   Gene   - Class in  bioroot      Gene()   - 
Constructor for class bioroot. Gene 
  
   GeneBean   - Class in  bioroot      GeneBean()   - 
Constructor for class bioroot. GeneBean 
  
   GeneBean(int, DBUtil)   - 
Constructor for class bioroot. GeneBean 
  
   GeneCentricCNVParser   - Class in  util.apps      GeneCentricCNVParser(String[])   - 
Constructor for class util.apps. GeneCentricCNVParser 
  
   GeneGroup   - Class in  util.bio.annotation  A GeneGroup is the top organizing level of a "Gene".   GeneGroup(String, String, int, int, String, TransGroup[], int, String)   - 
Constructor for class util.bio.annotation. GeneGroup 
  
   GeneNameCounter   - Class in  util.apps  Counts the frequency of words in the master list, then assigns these frequencies to words in the test list.   GeneNameCounter()   - 
Constructor for class util.apps. GeneNameCounter 
  
   generateConservedSeqs()   - 
Method in class gata.plotter. ConservedSeqs 
  
   generateFragments()   - 
Method in class edu.utah.seq.data. Simulator 
 Pick points
   generateIntervalStats(Interval)   - 
Method in class trans.main. IntervalReportPrinter 
 Calculate stats on oligos from best window.
   GeneRep   - Class in  util.bio.annotation  A GeneRep resentation contains a genomic sequence designating a gene broken into various segments.   GeneRep(TransGroup[], GeneGroup)   - 
Constructor for class util.bio.annotation. GeneRep 
  
   GeneRepGlyph   - Class in  gata.geneGlyphs      GeneRepGlyph(GeneRep, GATAParams, AnnoSpecParams)   - 
Constructor for class gata.geneGlyphs. GeneRepGlyph 
  
   GenericFeature   - Class in  util.bio.annotation  Class to hold user defined gff items like CRMs, enhancers, unpublished single track notation.   GenericFeature(GffFeature)   - 
Constructor for class util.bio.annotation. GenericFeature 
  
   GenericFeature(Gff3Feature)   - 
Constructor for class util.bio.annotation. GenericFeature 
  
   GenericGlyph   - Class in  gata.geneGlyphs      GenericGlyph(GenericFeature)   - 
Constructor for class gata.geneGlyphs. GenericGlyph 
  
   GenomeBean   - Class in  cgar.beans  Specific genome build/ release info, ie NCBI35.1   GenomeBean()   - 
Constructor for class cgar.beans. GenomeBean 
  
   GenomeFASTASplitter   - Class in  util.bio.converters  Splits a whole genome fasta into different files, also rewrites the fasta header to just the first word following the >   GenomeFASTASplitter()   - 
Constructor for class util.bio.converters. GenomeFASTASplitter 
  
   GenomicRegion   - Class in  trans.graphics      GenomicRegion(String, int)   - 
Constructor for class trans.graphics. GenomicRegion 
  
   GenomicRegionGlyph   - Class in  trans.graphics      GenomicRegionGlyph(String, int)   - 
Constructor for class trans.graphics. GenomicRegionGlyph 
  
   geoMeanFloatArraysFlipped(float[][])   - 
Static method in class util.gen. Num 
 Takes the geometric mean of float[][] values to double[], [numChips][OligoValues], returns the values
 in log base 2.
   geometricMean(double[])   - 
Static method in class util.gen. Num 
 Geometric mean.
   geometricMean(float[][])   - 
Static method in class util.gen. Num 
 Takes a geometric average of float[][] values [numChips][OligoValues].
   geometricMeanRatio(float[][], float[][])   - 
Static method in class util.gen. Num 
 Returns the log2 ratio of each pair, takes a geometric mean of replicas.
   geometricMeanSkipZeros(float[][])   - 
Static method in class util.gen. Num 
 Takes a geometric average of  float[][] values to double[], averages repeats [numChips][OligoValues].
   getA()   - 
Method in class gata.main. GATAParams 
  
   getA()   - 
Method in class util.bio.parsers. BaseCount 
  
   getAAlignPanel()   - 
Method in class gata.main. GATAParams 
  
   getAbsoluteDifference()   - 
Method in class trans.main. WilcoxonSamplePair 
  
   getAdd1PSPM()   - 
Method in class meme. MemeMotif 
  
   getAddOneLLPSPMCutOffScore()   - 
Method in class meme. MotifSearchResult 
  
   getAffiliation()   - 
Method in class cgar.beans. LabGroupBean 
  
   getAlias()   - 
Method in class bioroot. ReagentBean 
  
   getAlias()   - 
Method in class expr. MiRNATarget 
  
   getAlias()   - 
Method in class util.bio.parsers.gff. Gff3Feature 
 Reserved Attribute.
   getAlignerPreferences()   - 
Method in class gata.aligner. AlignerInputPanel 
  
   getAlignerPrefReference()   - 
Method in class gata.aligner. AlignerInputPanel 
  
   getAlignFrameHeight()   - 
Method in class gata.plotter. AlignPanel 
  
   getAlignFrameWidth()   - 
Method in class gata.plotter. AlignPanel 
  
   getAlignment()   - 
Method in class util.bio.wrappers. BlastResult 
  
   getAlignmentEnd()   - 
Method in class edu.utah.seq.methylation454. Read 
  
   getAlignments()   - 
Method in class gata.plotter. AlignPanel 
  
   getAlignmentStart()   - 
Method in class edu.utah.seq.methylation454. Read 
  
   getAlignmentString()   - 
Method in class gata.aligner. Alignment 
  
   getAlignPanel()   - 
Method in class gata.main. GATAParams 
  
   getAlignParams()   - 
Method in class gata.main. GATAParams 
  
   getAlignScrollPane()   - 
Method in class gata.main. GATAPlotter 
  
   getAmplicon()   - 
Method in class edu.utah.seq.methylation454. DataLine 
  
   getAmplicons()   - 
Method in class edu.utah.seq.methylation454. Sample 
  
   getAnnoPanelHeight()   - 
Method in class gata.main. AnnoSpecParams 
  
   getAnnotation()   - 
Method in class gata.main. AnnoSpecParams 
  
   getAntibodyForm(AntibodyBean, DBUtil, UserBean)   - 
Static method in class bioroot.antibody. AntibodyBase 
 Returns an antibody form filled with the values in the  bean.
   getAntibodyId()   - 
Method in class bioroot.antibody. AntibodyPrep 
  
   getAntibodyPreference()   - 
Method in class bioroot. UserBean 
  
   getAntibodyPrefs()   - 
Method in class bioroot. UserBean 
  
   getAntibodyPrepForm(AntibodyPrep, DBUtil, StringBuffer, int)   - 
Static method in class bioroot.antibody. AntibodyBase 
  
   getAntibodyPrepForms(AntibodyBean, DBUtil, StringBuffer)   - 
Static method in class bioroot.antibody. AntibodyBase 
  
   getAntibodyPrepReport(AntibodyPrep, DBUtil, StringBuffer, int, boolean)   - 
Static method in class bioroot.antibody. AntibodyBase 
  
   getAntibodyPrepReports(AntibodyBean, DBUtil, StringBuffer, boolean)   - 
Static method in class bioroot.antibody. AntibodyBase 
  
   getAntibodyPreps(DBUtil)   - 
Method in class bioroot.antibody. AntibodyBean 
  
   getAntibodyReport(AntibodyBean, DBUtil, UserBean, boolean)   - 
Static method in class bioroot.antibody. AntibodyBase 
 Returns an antibody report filled with the values in the  bean.
   getAntibodyUse()   - 
Method in class bioroot.antibody. UseDilution 
  
   getAntigen()   - 
Method in class bioroot.antibody. AntibodyBean 
  
   getAnyGenericGlyphs()   - 
Method in class gata.main. AnnoSpecParams 
  
   getAP()   - 
Method in class gata.aligner. LocalAlignment 
  
   getAppDescription()   - 
Method in class util.apps.gump. Gump 
  
   getAppDescriptionS()   - 
Method in class util.apps.gump. Gump 
  
   getApplicationPanel()   - 
Method in class util.apps.gump. GumpDrawFrame 
  
   getAppName()   - 
Method in class util.apps.gump. Gump 
  
   getAppsDirectory()   - 
Method in class trans.main. T2Parameter 
  
   getARFA()   - 
Method in class util.gen. SpearmanCorrelation 
  
   getArrayType()   - 
Method in class bioroot.strain. StrainBean 
  
   getAttributes()   - 
Method in class util.bio.annotation. GeneGroup 
  
   getAttributes()   - 
Method in class util.bio.annotation. GenericFeature 
  
   getAttributes()   - 
Method in class util.bio.parsers.gff. Gff3Feature 
  
   getAttsHash()   - 
Method in class util.bio.parsers.gff. GffFeature 
  
   getAttsString()   - 
Method in class util.bio.parsers.gff. GffFeature 
  
   getAvail()   - 
Method in class util.bio.digest. Enzyme 
  
   getAvailability()   - 
Method in class bioroot. ReagentBean 
  
   getAveExpSeqLength()   - 
Method in class selex. SelexParams 
  
   getAveNumExpSubSeqs()   - 
Method in class selex. SelexParams 
  
   getAveNumObsSubSeqs()   - 
Method in class selex. SelexParams 
  
   getAveObsExpRatio()   - 
Method in class selex. SelexParams 
  
   getAverageIntensityValues()   - 
Method in class trans.main. WilcoxonSignedRankTest 
  
   getAverageInts(int, int)   - 
Static method in class util.gen. Num 
 Gets the average of the integers bracketed and including the start and end.
   getAwsCredentials()   - 
Method in class util.amazon. S3Manager 
  
   getB()   - 
Method in class gata.main. GATAParams 
  
   getBackgroundColor()   - 
Method in class gata.main. GATAParams 
  
   getBarFile()   - 
Method in class edu.utah.seq.parsers. BarParser 
  
   getBarParser()   - 
Method in class edu.utah.seq.data. PointData 
  
   getBase()   - 
Method in class util.bio.parsers. BaseCount 
  
   getBaseName()   - 
Method in class gata.aligner. AlignerPreferences 
  
   getBaseName()   - 
Method in class gata.aligner. AlignParams 
  
   getBaseNumArray(String, int)   - 
Static method in class gata.main. GATAUtil 
  
   getBasePositions()   - 
Method in class edu.utah.seq.parsers. BarParser 
  
   getBasePositions()   - 
Method in class trans.misc. GrGraph 
  
   getBaseScore()   - 
Method in class edu.utah.seq.data. HeatMapMaker 
  
   getBaseScore()   - 
Method in class trans.main. WindowBlockMaker 
  
   getBaseScores()   - 
Method in class meme. MotifScanner 
  
   getBaseScores()   - 
Method in class trans.main. Interval 
  
   getBasicTm(String)   - 
Method in class bioroot.oligo. OligoBean 
  
   getBathrooms()   - 
Method in class util.apps. HouseBean 
  
   getBedrooms()   - 
Method in class util.apps. HouseBean 
  
   getBestSubWindow()   - 
Method in class edu.utah.seq.data. EnrichedRegion 
  
   getBestSubWindow()   - 
Method in class trans.main. Interval 
  
   getBestSubWindowSequence()   - 
Method in class trans.main. Interval 
  
   getBestWindow()   - 
Method in class edu.utah.seq.data. EnrichedRegion 
  
   getBestWindow()   - 
Method in class trans.main. Interval 
  
   getBestWindowSequence()   - 
Method in class trans.main. Interval 
  
   getBiggestAlignPanelY()   - 
Method in class gata.main. GATAParams 
  
   getBiggestGlyphPanelY()   - 
Method in class gata.main. AnnoSpecParams 
  
   getBinCounts()   - 
Method in class util.gen. Histogram 
  
   getBindingPeaks()   - 
Method in class trans.main. Interval 
  
   getBinLabels()   - 
Method in class util.gen. Histogram 
  
   getBioroot()   - 
Method in class bioroot. UserRegistrationBean 
  
   getBitScore()   - 
Method in class gata.aligner. Alignment 
  
   getBl2F()   - 
Method in class gata.aligner. AlignerInputPanel 
  
   getBl2seq()   - 
Method in class gata.aligner. AlignParams 
  
   getBl2SeqHits()   - 
Method in class bioroot. BlastMatcher 
  
   getBl2SeqProg()   - 
Method in class gata.aligner. AlignerPreferences 
  
   getBlastResults()   - 
Method in class bioroot. SearchBean 
  
   getBooleans()   - 
Method in class util.bio.seq. BinaryBooleanReaderWriter 
  
   getBottomCluster()   - 
Method in class util.bio.cluster. Cluster 
  
   getBoundingBox()   - 
Method in class gata.geneGlyphs. GeneRepGlyph 
  
   getBoundingBox()   - 
Method in class gata.geneGlyphs. GenericGlyph 
  
   getBpMatches()   - 
Method in class bioroot.plasmid. OligoMatch 
  
   getBRFA()   - 
Method in class util.gen. SpearmanCorrelation 
  
   getBucket()   - 
Method in class util.amazon. S3Manager 
  
   getBucketName()   - 
Method in class util.amazon. S3Manager 
  
   getC()   - 
Method in class gata.main. GATAParams 
  
   getC()   - 
Method in class util.bio.parsers. BaseCount 
  
   getCdsEnd()   - 
Method in class util.bio.parsers. UCSCGeneLine 
  
   getCdsStart()   - 
Method in class util.bio.parsers. UCSCGeneLine 
  
   getCelFiles()   - 
Method in class trans.main. Interval 
  
   getCell(String)   - 
Method in class bioroot. DBUtil 
 Returns the first cell from an sql statement.
   getCell(String)   - 
Method in class util.gen. SQL 
 Returns the first cell from an sql statement.
   getCellTypeId(String, int)   - 
Method in class bioroot. DBUtil 
 Fetches an tissueId given a tissue name and labGroupId (they aren't unique except within a labGroup)
   getCellTypeId()   - 
Method in class bioroot.strain. StrainBean 
  
   getCellTypeName(DBUtil)   - 
Method in class bioroot.strain. StrainBean 
 Will look one time for a name, all other times will just return "".
   getCelMasker()   - 
Method in class trans.graphics. VirtualCelFrame 
  
   getCelpFiles()   - 
Method in class trans.cel. QuantileNormalization 
  
   getChallenge()   - 
Method in class bioroot. LoginBean 
  
   getChallengeConcatinate()   - 
Method in class util.gen. Passwords 
  
   getChallengeDate()   - 
Method in class bioroot. LoginBean 
  
   getChart()   - 
Method in class trans.graphics. RankedSetAnalysis 
  
   getChartPanel()   - 
Method in class trans.graphics. RankedSetAnalysis 
  
   getCheckBoxNumber()   - 
Method in class bioroot. Feature 
 Makes a checkbox and textfield where the box is checked if Feature is visible and a rounded double
 orderNumber is put in text field if it's not 1000
   getCheckBoxNumber(String)   - 
Method in class bioroot. TableHeading 
 Makes a checkbox and textfield where the box is checked if TableHeading is visible and a rounded double
 orderNumber is put in text field if it's not 1000
   getChipSetDirectories()   - 
Method in class trans.main. ChromSet 
  
   getChrom()   - 
Method in class util.bio.parsers. UCSCGeneLine 
  
   getChromosome()   - 
Method in class edu.utah.seq.data. EnrichedRegion 
  
   getChromosome()   - 
Method in class edu.utah.seq.data. Info 
  
   getChromosome()   - 
Method in class edu.utah.seq.methylation454. Reference 
  
   getChromosome()   - 
Method in class edu.utah.seq.parsers. BarParser 
  
   getChromosome()   - 
Method in class edu.utah.seq.parsers. ElandExportLine 
  
   getChromosome()   - 
Method in class expr. CorrelationWindow 
  
   getChromosome()   - 
Method in class trans.anno. BindingRegion 
  
   getChromosome()   - 
Method in class trans.anno. Region 
  
   getChromosome()   - 
Method in class trans.bkgrnd. LoadedOligo 
  
   getChromosome()   - 
Method in class trans.cel. ChromosomeIndex 
  
   getChromosome()   - 
Method in class trans.graphics. GenomicRegion 
  
   getChromosome()   - 
Method in class trans.main. Interval 
  
   getChromosome()   - 
Method in class trans.main. PosRegion 
  
   getChromosome()   - 
Method in class trans.main. Window 
  
   getChromosome()   - 
Method in class trans.misc. GrGraph 
  
   getChromosome()   - 
Method in class trans.roc. ControlGene 
  
   getChromosome()   - 
Method in class trans.roc. Positive 
  
   getChromosome()   - 
Method in class trans.roc. RocWindow 
  
   getChromosome()   - 
Method in class trans.roc. Sgr 
  
   getChromosome()   - 
Method in class trans.tpmap. BlastFilterResult 
  
   getChromosome()   - 
Method in class trans.tpmap. TPMapFeature 
  
   getChromosome()   - 
Method in class trans.tpmap. TPMapLine 
  
   getChromosome()   - 
Method in class util.bio.annotation. Coordinate 
  
   getChromosome()   - 
Method in class util.bio.annotation. GeneGroup 
  
   getChromosome()   - 
Method in class util.bio.seq. IndexedSequence 
  
   getChromosomeNames()   - 
Method in class cgar.beans. GenomeBean 
  
   getChromosomeNumber()   - 
Static method in class trans.misc. Util 
 Creates a HashMap of "chr1"=Byte(1) through "chr22"=Byte(22) plus 
 chrX=23, chrY=24, and chrMT=25.
   getChromSpecificGeneLines()   - 
Method in class util.bio.parsers. UCSCGeneModelTableReader 
  
   getChromSplitFeatures()   - 
Method in class util.bio.parsers.gff. Gff3Parser 
 Returns the features split by chromosome.
   getCity()   - 
Method in class util.apps. HouseBean 
  
   getClosestBindingRegion()   - 
Method in class trans.anno. BindingRegion 
  
   getClosestInterval()   - 
Method in class trans.main. PosRegion 
  
   getClosestNonOverlappingInterval()   - 
Method in class trans.main. PosRegion 
  
   getClusters()   - 
Method in class util.bio.cluster. HierarchicalClustering 
  
   getCmdLn()   - 
Method in class meme. MemeParser 
  
   getCodingSegments()   - 
Method in class util.bio.annotation. GeneRep 
 Conservative estimate of coding segments.
   getCodingSegments()   - 
Method in class util.bio.annotation. Translation 
  
   getCoefficientOfVariation()   - 
Method in class trans.qc. CelFileStats 
  
   getCollapsed()   - 
Method in class bioroot. IdNameMatch 
  
   getColor()   - 
Method in class gata.geneGlyphs. GenericGlyph 
  
   getColumnName()   - 
Method in class bioroot. Feature 
  
   getColumnName()   - 
Method in class bioroot. SearchHit 
  
   getColumnText()   - 
Method in class bioroot. SearchHit 
  
   getCommandArray()   - 
Method in class util.bio.wrappers. BL2Seq 
  
   getComments()   - 
Method in class bioroot. ReagentBean 
  
   getCompAnnoSpecParams()   - 
Method in class gata.main. GATAParams 
  
   getCompScrollPane()   - 
Method in class gata.main. GATAPlotter 
  
   getCompSeq()   - 
Method in class gata.aligner. AlignerPreferences 
  
   getCompSeqFile()   - 
Method in class gata.aligner. AlignParams 
  
   getCompStart()   - 
Method in class gata.aligner. AlignerPreferences 
  
   getCompTexF()   - 
Method in class gata.aligner. AlignerInputPanel 
  
   getConcatStart()   - 
Method in class bioroot. BlastMatcher 
  
   getConcatStop()   - 
Method in class bioroot. BlastMatcher 
  
   getConcentration()   - 
Method in class bioroot.oligo. OligoBean 
  
   getConcentration()   - 
Method in class bioroot.plasmid. PlasmidBean 
  
   getConcentrationUnits()   - 
Method in class bioroot.oligo. OligoBean 
  
   getConnection()   - 
Method in class bioroot. DBUtil 
  
   getConnection()   - 
Method in class util.gen. SQL 
  
   getConsole()   - 
Method in class gata.main. GATAParams 
  
   getConsole()   - 
Method in class gata.plotter. ConservedSeqs 
  
   getContactEmail()   - 
Method in class bioroot. ContactBean 
  
   getContainingGeneGrps()   - 
Method in class trans.anno. BindingRegion 
  
   getControlCelFiles()   - 
Method in class trans.main. T2Parameter 
  
   getControlIntensities(int)   - 
Method in class trans.main. Oligo 
  
   getControlIntensities()   - 
Method in class trans.main. ScanChromosomes 
  
   getControlIntensities()   - 
Method in class trans.main. ScanChromosomesCNV 
  
   getControlPoints()   - 
Method in class edu.utah.seq.data. EnrichedRegion 
  
   getControlStats()   - 
Method in class trans.cel. QuantileNormalization 
  
   getControlValues()   - 
Method in class trans.main. ScoredGene 
  
   getCoordinates()   - 
Method in class expr. ExpressedGene 
  
   getCoordinates()   - 
Method in class gata.aligner. Alignment 
  
   getCoordinates()   - 
Method in class trans.bkgrnd. BackGroundOligo 
  
   getCorrelationCoefficient()   - 
Method in class util.bio.cluster. Cluster 
  
   getCpG()   - 
Method in class edu.utah.seq.methylation454. DataLine 
  
   getCpGGenomicPositions()   - 
Method in class edu.utah.seq.methylation454. Reference 
  
   getCpGPositions()   - 
Method in class edu.utah.seq.methylation454. Reference 
  
   getCpGs()   - 
Method in class edu.utah.seq.methylation454. Amplicon 
  
   getCreate()   - 
Method in class gata.aligner. AlignerPreferences 
  
   getCredentialsFile()   - 
Method in class util.amazon. S3Manager 
  
   getCustomAttributes()   - 
Method in class util.bio.parsers.gff. Gff3Feature 
 Any Attribute that is not a reserve attribute is grouped here.
   getCutBottom()   - 
Method in class util.bio.digest. Enzyme 
  
   getCutTop()   - 
Method in class util.bio.digest. Enzyme 
  
   getCValue()   - 
Method in class trans.main. WilcoxonSamplePair 
  
   getD()   - 
Method in class gata.main. GATAParams 
  
   getDashStroke()   - 
Method in class gata.main. GATAParams 
  
   getDate()   - 
Method in class bioroot.antibody. AntibodyPrep 
  
   getDate()   - 
Method in class cgar.beans. AnalysisBean 
  
   getDate()   - 
Static method in class util.gen. Misc 
 Returns a nicely formated time (15 May 2004).
   getDate()   - 
Method in class util.gen. Passwords 
  
   getDateLastLogin()   - 
Method in class cgar.beans. UserBean 
  
   getDateNoSpaces()   - 
Static method in class util.gen. Misc 
 Returns a nicely formated time (15May2004).
   getDbxref()   - 
Method in class util.bio.parsers.gff. Gff3Feature 
 Reserved Attribute.
   getDefaultTrackThickness()   - 
Method in class gata.main. GATAParams 
  
   getDeletions()   - 
Method in class util.bio.parsers. BaseCount 
  
   getDemoResponse()   - 
Method in class bioroot. LoginBean 
  
   getDescription()   - 
Method in class cgar.beans. AnalysisBean 
  
   getDescription()   - 
Method in class cgar.beans. AnalysisGroupBean 
  
   getDescription()   - 
Method in class cgar.beans. AnalysisTypeBean 
  
   getDescription()   - 
Method in class cgar.beans. ProcessedDataTypeBean 
  
   getDescription()   - 
Method in class util.apps.gump. Gump 
  
   getDescription()   - 
Method in class util.apps.gump. Option 
  
   getDescriptionS()   - 
Method in class util.apps.gump. Option 
  
   getDifference()   - 
Method in class trans.main. WilcoxonSamplePair 
  
   getDifferentUserConcat()   - 
Method in class bioroot. UserRegistrationBean 
  
   getDilution()   - 
Method in class bioroot.antibody. UseDilution 
  
   getDisplayName()   - 
Method in class util.bio.parsers. UCSCGeneLine 
  
   getDistanceTo3PrimeGeneGrp()   - 
Method in class trans.anno. BindingRegion 
  
   getDistanceTo5PrimeGeneGrp()   - 
Method in class trans.anno. BindingRegion 
  
   getDNAColor()   - 
Method in class gata.main. GATAParams 
  
   getDNAFILES()   - 
Method in class gata.aligner. AlignParams 
  
   getDocs()   - 
Static method in class meme. UtilMeme 
  
   getDottedProteinLines()   - 
Method in class gata.main. AnnoSpecParams 
  
   getDUST()   - 
Method in class gata.aligner. AlignParams 
  
   getE()   - 
Method in class gata.main. GATAParams 
  
   getEditHistory()   - 
Method in class bioroot. CellTypeBean 
  
   getEditHistory()   - 
Method in class bioroot. GeneBean 
  
   getEditHistory()   - 
Method in class bioroot. MarkerBean 
  
   getEditHistory()   - 
Method in class bioroot. OrganismBean 
  
   getEditHistory()   - 
Method in class bioroot. ReagentBean 
  
   getEditHistory()   - 
Method in class bioroot. UserBean 
  
   getEFF_M()   - 
Method in class gata.aligner. AlignParams 
  
   getEFF_N()   - 
Method in class gata.aligner. AlignParams 
  
   getElementValue(Node)   - 
Static method in class util.xml. ParseXMLFile 
 Returns element value
   getEmail()   - 
Method in class bioroot. UserBean 
  
   getEmail()   - 
Method in class bioroot. UserRegistrationBean 
  
   getEmail()   - 
Method in class cgar.beans. UserBean 
  
   getEmailAddresses()   - 
Method in class bioroot. LabGroup 
  
   getEmailObject()   - 
Static method in class bioroot. Contact 
 Returns an Email object for posting email.
   getEmailObject()   - 
Static method in class bioroot. Login 
 Returns an Email object for posting email.
   getEnd()   - 
Method in class gata.geneGlyphs. GeneRepGlyph 
  
   getEnd()   - 
Method in class trans.anno. BindingRegion 
  
   getEnd()   - 
Method in class trans.anno. Region 
  
   getEnd()   - 
Method in class trans.main. PosRegion 
  
   getEnd()   - 
Method in class trans.roc. RocWindow 
  
   getEnd()   - 
Method in class util.bio.annotation. ExonIntron 
  
   getEnd()   - 
Method in class util.bio.annotation. GeneGroup 
  
   getEnd()   - 
Method in class util.bio.annotation. GenericFeature 
  
   getEnd()   - 
Method in class util.bio.annotation. StartStop 
  
   getEnd()   - 
Method in class util.bio.annotation. Transcript 
  
   getEnd()   - 
Method in class util.bio.annotation. Translation 
  
   getEnd()   - 
Method in class util.bio.parsers.gff. Gff3Feature 
  
   getEnd()   - 
Method in class util.bio.parsers.gff. GffFeature 
  
   getEnzymes()   - 
Method in class util.bio.digest. NEBParser 
  
   getExactMatches()   - 
Method in class trans.tpmap. BlastFilterResult 
  
   getExonEnds()   - 
Method in class trans.roc. ControlGene 
  
   getExons()   - 
Method in class util.bio.annotation. TransGroup 
  
   getExons()   - 
Method in class util.bio.parsers. UCSCGeneLine 
  
   getExonStarts()   - 
Method in class trans.roc. ControlGene 
  
   getExpect()   - 
Method in class util.bio.wrappers. BlastResult 
  
   getExpectation()   - 
Method in class util.bio.wrappers. BL2Seq 
  
   getExpectedSize()   - 
Method in class selex. SelexParams 
  
   getExtend()   - 
Method in class gata.aligner. AlignerPreferences 
  
   getExtract()   - 
Method in class gata.aligner. AlignerPreferences 
  
   getExtractedExons()   - 
Method in class util.bio.annotation. TransGroup 
  
   getF()   - 
Method in class gata.main. GATAParams 
  
   getFailedLogins()   - 
Method in class bioroot. UserBean 
  
   getFamilyRooms()   - 
Method in class util.apps. HouseBean 
  
   getFastas()   - 
Method in class util.bio.parsers. MultiFastaParser 
  
   getFatLineThicknes()   - 
Method in class gata.main. GATAParams 
  
   getFatRNALines()   - 
Method in class gata.main. AnnoSpecParams 
  
   getFatStroke()   - 
Method in class gata.main. GATAParams 
  
   getFdr()   - 
Method in class trans.roc. Bin 
  
   getFeature()   - 
Method in class util.bio.parsers.gff. GffFeature 
  
   getFeatureNames(Feature[])   - 
Static method in class bioroot. Feature 
  
   getFeatures()   - 
Method in class bioroot. Preference 
  
   getFeatures()   - 
Method in class util.bio.parsers.gff. Gff3Parser 
  
   getFeatureType()   - 
Method in class util.bio.annotation. GenericFeature 
  
   getFieldGroup()   - 
Method in class bioroot. SearchBean 
  
   getFields()   - 
Method in class bioroot. SearchBean 
  
   getFileName()   - 
Method in class bioroot.antibody. AntibodyBean 
  
   getFileName()   - 
Method in class bioroot.antibody. AntibodyPrep 
  
   getFileName()   - 
Method in class bioroot.oligo. OligoUploadBean 
  
   getFileName()   - 
Method in class bioroot.plasmid. PlasmidBean 
  
   getFileName()   - 
Method in class bioroot.plasmid. PlasmidUploadBean 
  
   getFileName()   - 
Method in class bioroot.strain. StrainBean 
  
   getFileName()   - 
Method in class bioroot.strain. StrainUploadBean 
  
   getFileName()   - 
Method in class util.bio.parsers. QualityFileParser 
  
   getFileNumber()   - 
Method in class meme. MotifSearchResult 
  
   getFirstName()   - 
Method in class bioroot. UserBean 
  
   getFirstName()   - 
Method in class bioroot. UserRegistrationBean 
  
   getFirstName()   - 
Method in class cgar.beans. UserBean 
  
   getFirstPeakIndex()   - 
Method in class trans.main. BindingPeak 
  
   getFivePrimeNonCodingRegion()   - 
Method in class util.bio.annotation. GeneRep 
 Conservative estimate returning smallest non coding region.
   getFlag()   - 
Method in class util.apps.gump. Option 
  
   getFlagS()   - 
Method in class util.apps.gump. Option 
  
   getFloatArrayFile()   - 
Method in class util.bio.cluster. Cluster 
  
   getFont()   - 
Method in class gata.main. GATAParams 
  
   getFormattedTimeFromFraction(double)   - 
Static method in class util.gen. Misc 
 Returns a formatted time 2:15:00 given 2.25
   getFractionIntersections()   - 
Method in class trans.main. Interval 
  
   getFreqMatrix()   - 
Method in class meme. MemeMotif 
  
   getFullName()   - 
Method in class bioroot. TableHeading 
  
   getFullPathName(File)   - 
Static method in class util.gen. IO 
 Gets full path name using getCanonicalPath() on a File
   getFullPathToBL2seq()   - 
Method in class util.bio.wrappers. BL2Seq 
  
   getFurthestDown()   - 
Method in class gata.aligner. AlignerInputPanel 
  
   getFurthestRight()   - 
Method in class gata.aligner. AlignerInputPanel 
  
   getG()   - 
Method in class util.bio.parsers. BaseCount 
  
   getGap()   - 
Method in class util.bio.parsers.gff. Gff3Feature 
 Reserved Attribute.
   getGAP_CREATE()   - 
Method in class gata.aligner. AlignParams 
  
   getGAP_EXT()   - 
Method in class gata.aligner. AlignParams 
  
   getGaps()   - 
Method in class util.bio.wrappers. BlastResult 
  
   getGarage()   - 
Method in class util.apps. HouseBean 
  
   getGataFrame()   - 
Method in class gata.main. GATAParams 
  
   getGataPlotter()   - 
Method in class gata.main. GATAParams 
  
   getGC(String)   - 
Method in class bioroot.oligo. OligoBean 
  
   getGc()   - 
Method in class trans.bkgrnd. LoadedOligo 
  
   getGc()   - 
Method in class trans.misc. GCRatio 
  
   getGcContent()   - 
Method in class trans.anno. BindingRegion 
  
   getGcContent()   - 
Method in class trans.anno. Region 
  
   getGene()   - 
Method in class trans.main. ScoredGene 
  
   getGeneGlyphs()   - 
Method in class gata.main. AnnoSpecParams 
  
   getGeneGroupArrayList()   - 
Method in class util.bio.parsers.gff. DmelRel4Extractor 
  
   getGeneGroups()   - 
Method in class util.bio.parsers.gff. DmelRel4Extractor 
 Final product from the extractor.
   getGeneGrp()   - 
Method in class util.bio.annotation. GeneRep 
  
   getGeneGrp3Prime()   - 
Method in class trans.anno. BindingRegion 
  
   getGeneGrp5Prime()   - 
Method in class trans.anno. BindingRegion 
  
   getGeneGrps()   - 
Method in class util.bio.parsers.gff. GadFlyGffExtractor 
  
   getGeneId(DBUtil)   - 
Method in class bioroot.antibody. AntibodyBean 
  
   getGeneId(String, int)   - 
Method in class bioroot. DBUtil 
 Fetches an geneId given a geneName and labGroupId (they aren't unique except within a labGroup),
  assumes connection to BioRoot+Database.
   getGeneId()   - 
Method in class bioroot.oligo. OligoBean 
  
   getGeneId()   - 
Method in class bioroot.plasmid. PlasmidBean 
  
   getGeneLines()   - 
Method in class util.bio.parsers. UCSCGeneModelTableReader 
  
   getGeneName(DBUtil)   - 
Method in class bioroot.antibody. AntibodyBean 
  
   getGeneName(int)   - 
Method in class bioroot. DBUtil 
 Fetches an geneName given an geneId, assumes connection to BioRoot+Database.
   getGeneName(DBUtil)   - 
Method in class bioroot.oligo. OligoBean 
  
   getGeneName(DBUtil)   - 
Method in class bioroot.plasmid. PlasmidBean 
  
   getGeneRep()   - 
Method in class util.bio.annotation. GeneGroup 
  
   getGenericFeature()   - 
Method in class gata.geneGlyphs. GenericGlyph 
  
   getGenericFeatureHash()   - 
Method in class util.bio.parsers.gff. DmelRel4Extractor 
 Contains all the feature types that were not recognized by the Extractor.
   getGenericFeatureHash()   - 
Method in class util.bio.parsers.gff. GadFlyGffExtractor 
 Contains all the feature types that were not recognized by the GadFlyGFFExtractor.
   getGenericFeatures()   - 
Method in class util.bio.parsers.gff. DmelRel4Extractor 
  
   getGenericFeatures()   - 
Method in class util.bio.parsers.gff. GadFlyGffExtractor 
  
   getGenericFeaturesAL()   - 
Method in class util.bio.parsers.gff. DmelRel4Extractor 
  
   getGenericFeaturesHash()   - 
Method in class util.bio.parsers.gff. DmelRel4Extractor 
  
   getGenericVisibility()   - 
Method in class bioroot. ReagentBean 
 Returns one of the standard visibility settings (WWW, Lab mates, Private) or the generic 'Organization'
   getGeneSequence()   - 
Method in class bioroot.oligo. OligoBean 
  
   getGenomeFastaDirectory()   - 
Method in class trans.main. T2Parameter 
  
   getGenomeId()   - 
Method in class cgar.beans. AnalysisBean 
  
   getGenomeVersion()   - 
Method in class trans.main. T2Parameter 
  
   getGenomeVersion()   - 
Method in class trans.misc. GrGraph 
  
   getGenotype()   - 
Method in class bioroot.strain. StrainBean 
  
   getGffFeatures()   - 
Method in class util.bio.parsers.gff. GffParser 
  
   getGffFile()   - 
Method in class gata.main. AnnoSpecParams 
  
   getGFFFile()   - 
Method in class util.bio.parsers.gff. Gff3Parser 
  
   getGffLine()   - 
Method in class util.bio.parsers.gff. GffFeature 
  
   getGlyph()   - 
Method in class trans.graphics. GenomicRegion 
  
   getGlyphPanel()   - 
Method in class gata.geneGlyphs. Annotation 
  
   getGlyphPanel()   - 
Method in class gata.main. AnnoSpecParams 
  
   getGraphics2DN()   - 
Method in class gata.main. AnnoSpecParams 
  
   getGroupName()   - 
Method in class trans.qc. CelFileStats 
  
   getGrowthConditions()   - 
Method in class bioroot.strain. StrainBean 
  
   getH()   - 
Method in class gata.aligner. AlignParams 
  
   getHandlingInstructions()   - 
Method in class bioroot.oligo. OligoBean 
  
   getHeader(int, int)   - 
Static method in class trans.main. ExportIntervalData 
  
   getHeader()   - 
Method in class util.bio.digest. NEBParser 
  
   getHeader()   - 
Static method in class util.bio.wrappers. Primer3Pick 
  
   getHeight()   - 
Method in class gata.main. GATAParams 
  
   getHeight()   - 
Method in class util.apps.gump. GumpDrawFrame 
  
   getHeightBuffImage()   - 
Method in class gata.plotter. AlignPanel 
  
   getHeightLabels()   - 
Method in class gata.main. GATAParams 
  
   getHits()   - 
Method in class meme. MemeMotif 
  
   getHits()   - 
Method in class trans.anno. Region 
  
   getHits()   - 
Method in class util.bio.wrappers. BL2Seq 
  
   getHits()   - 
Method in class util.gen. Bin 
  
   getHitsAddOneLLPSPM()   - 
Method in class meme. MotifSearchResult 
  
   getHitsMemeLLPSPM()   - 
Method in class meme. MotifSearchResult 
  
   getHitsPerRound()   - 
Method in class util.bio.xamer. Xamer 
  
   getHotLinkedOligos()   - 
Method in class bioroot.plasmid. PlasmidBean 
 Returns HTML for of oligoMatches for display.
   getHotLinkedParents(DBUtil)   - 
Method in class bioroot.strain. StrainBean 
 Returns HTML parent/ strain names for display.
   getHotLinkedPlasmids(DBUtil)   - 
Method in class bioroot.antibody. AntibodyBean 
 Returns HTML plasmid names for display.
   getHotLinkedPlasmids(DBUtil)   - 
Method in class bioroot.strain. StrainBean 
 Returns HTML plasmid names for display.
   getHotLinkedStrains(DBUtil)   - 
Method in class bioroot.antibody. AntibodyBean 
 Returns HTML strain names for display.
   getHotLinkedStrains(DBUtil)   - 
Method in class bioroot.plasmid. PlasmidBean 
 Returns HTML strain names for display.
   getHtmlChallengeTable()   - 
Method in class util.gen. Passwords 
  
   getId()   - 
Method in class bioroot.antibody. AntibodyPrep 
  
   getId()   - 
Method in class bioroot.antibody. UseDilution 
  
   getId()   - 
Method in class bioroot. BlastMatcher 
  
   getId()   - 
Method in class bioroot. CellTypeBean 
  
   getId()   - 
Method in class bioroot. ContactBean 
  
   getId()   - 
Method in class bioroot. GeneBean 
  
   getId()   - 
Method in class bioroot. IdNameMatch 
  
   getId()   - 
Method in class bioroot. LabGroup 
  
   getId()   - 
Method in class bioroot. MarkerBean 
  
   getId()   - 
Method in class bioroot. OrganismBean 
  
   getId()   - 
Method in class bioroot. Organization 
  
   getId()   - 
Method in class bioroot.plasmid. OligoMatch 
  
   getId()   - 
Method in class bioroot. ReagentBean 
  
   getId()   - 
Method in class bioroot. SearchHit 
  
   getId()   - 
Method in class bioroot. UserBean 
  
   getId()   - 
Method in class cgar.beans. AnalysisBean 
  
   getId()   - 
Method in class cgar.beans. AnalysisGroupBean 
  
   getId()   - 
Method in class cgar.beans. AnalysisTypeBean 
  
   getId()   - 
Method in class cgar.beans. GenomeBean 
  
   getId()   - 
Method in class cgar.beans. LabGroupBean 
  
   getId()   - 
Method in class cgar.beans. ProcessedDataBean 
  
   getId()   - 
Method in class cgar.beans. ProcessedDataTypeBean 
  
   getId()   - 
Method in class cgar.beans. RawDataBean 
  
   getId()   - 
Method in class cgar.beans. SpeciesBean 
  
   getId()   - 
Method in class cgar.beans. UserBean 
  
   getId()   - 
Method in class cgar.beans. UserTypeBean 
  
   getId()   - 
Method in class cgar.beans. VisibilityBean 
  
   getId()   - 
Method in class edu.utah.seq.methylation454. Amplicon 
  
   getId()   - 
Method in class edu.utah.seq.methylation454. BaseRead 
  
   getId()   - 
Method in class edu.utah.seq.methylation454. CpG 
  
   getId()   - 
Method in class edu.utah.seq.methylation454. Sample 
  
   getId()   - 
Method in class util.bio.parsers.gff. Gff3Feature 
 Reserved Attribute.
   getIdentities()   - 
Method in class util.bio.wrappers. BlastResult 
  
   getIncrement()   - 
Method in class util.gen. Histogram 
  
   getIndex()   - 
Method in class trans.main. Oligo 
  
   getIndex()   - 
Method in class trans.main. SubWindow 
  
   getInfo()   - 
Method in class edu.utah.seq.data. InfoPoint 
  
   getInfo()   - 
Method in class edu.utah.seq.data. PointData 
  
   getInfo()   - 
Method in class edu.utah.seq.data. SmoothingWindowInfo 
  
   getInnerSplitPane()   - 
Method in class gata.main. GATAPlotter 
  
   getInsertionLengths()   - 
Method in class util.bio.parsers. BaseCount 
  
   getIntensities()   - 
Method in class trans.cel. QuantileNormalization 
  
   getIntensities()   - 
Method in class trans.main. Oligo 
  
   getIntensities()   - 
Method in class trans.qc. CelFileStats 
  
   getIntensities()   - 
Method in class trans.roc. ControlGene 
  
   getIntensity()   - 
Method in class trans.tpmap. TPMapFeature 
  
   getIntensityArrayLists()   - 
Method in class trans.bkgrnd. BackGroundOligo 
  
   getIntensityMM()   - 
Method in class trans.bkgrnd. LoadedOligo 
  
   getIntensityPM()   - 
Method in class trans.bkgrnd. LoadedOligo 
  
   getIntercept()   - 
Method in class util.gen. LinearRegression 
  
   getIntersectingRegions()   - 
Method in class trans.graphics. GenomicRegion 
  
   getInterval()   - 
Method in class trans.main. PeakPicker 
  
   getIntrons()   - 
Method in class util.bio.annotation. Translation 
  
   getIso()   - 
Method in class util.bio.digest. Enzyme 
  
   getJarFile()   - 
Method in class util.apps.gump. Gump 
  
   getJava()   - 
Method in class trans.main. T2Parameter 
  
   getJava()   - 
Method in class util.apps.gump. Gump 
  
   getK()   - 
Method in class gata.aligner. AlignParams 
  
   getLabel()   - 
Method in class trans.graphics. GenomicRegionGlyph 
  
   getLabel()   - 
Method in class util.bio.annotation. GeneGroup 
  
   getLabel()   - 
Method in class util.gen. Bin 
  
   getLabelColor()   - 
Method in class gata.main. GATAParams 
  
   getLabels()   - 
Method in class gata.main. AnnoSpecParams 
  
   getLabels()   - 
Method in class util.bio.cluster. ClusterDrawPanel 
  
   getLabGroup()   - 
Method in class bioroot. LoginBean 
  
   getLabGroup()   - 
Method in class bioroot. UserBean 
  
   getLabGroup()   - 
Method in class bioroot. UserRegistrationBean 
  
   getLabGroupId()   - 
Method in class bioroot. CellTypeBean 
  
   getLabGroupId()   - 
Method in class bioroot. GeneBean 
  
   getLabGroupId()   - 
Method in class bioroot. LoginBean 
  
   getLabGroupId()   - 
Method in class bioroot. ReagentBean 
  
   getLabGroupId()   - 
Method in class bioroot. UserBean 
  
   getLabGroupId()   - 
Method in class cgar.beans. AnalysisBean 
  
   getLabGroupId()   - 
Method in class cgar. LabGroupUserType 
  
   getLabGroupName()   - 
Method in class bioroot. ContactBean 
  
   getLabGroupName(DBUtil)   - 
Method in class bioroot. ReagentBean 
  
   getLabGroupName()   - 
Method in class cgar. LabGroupUserType 
  
   getLabGroups()   - 
Method in class bioroot. Preference 
  
   getLabGroupSchedule(DBUtil)   - 
Method in class bioroot. UserBean 
  
   getLabGroupsToArchive(DBUtil)   - 
Static method in class bioroot. CronArchive 
 Identifies lab groups that should be archived based on their last archive date and schedule.
   getLabGroupUserTypes()   - 
Method in class cgar.beans. UserBean 
  
   getLAMBDA()   - 
Method in class gata.aligner. AlignParams 
  
   getLAScore()   - 
Method in class gata.aligner. LocalAlignment 
  
   getLastInsertId(String)   - 
Method in class bioroot. DBUtil 
 Returns the last inserted row auto increment number.
   getLastInsertId(String)   - 
Method in class util.gen. SQL 
 Returns the last inserted row auto increment number.
   getLastLogin()   - 
Method in class bioroot. UserBean 
  
   getLastName()   - 
Method in class bioroot. UserBean 
  
   getLastName()   - 
Method in class bioroot. UserRegistrationBean 
  
   getLastName()   - 
Method in class cgar.beans. UserBean 
  
   getLastUser()   - 
Method in class bioroot.antibody. AntibodyPrep 
  
   getLastUser()   - 
Method in class bioroot. ReagentBean 
  
   getLatitude()   - 
Method in class util.apps. HouseBean 
  
   getLeftFlankingIndex()   - 
Method in class trans.main. BindingPeak 
  
   getLeftIndex()   - 
Method in class trans.main. PeakPicker 
  
   getLeftLR()   - 
Method in class trans.cel. SplineScalar 
  
   getLeftSideMatch()   - 
Method in class selex. SelexParams 
  
   getLeftSideReplace()   - 
Method in class selex. SelexParams 
  
   getLeftSlope()   - 
Method in class trans.main. Oligo 
  
   getLength()   - 
Method in class bioroot.oligo. OligoBean 
  
   getLength()   - 
Method in class bioroot.plasmid. PlasmidBean 
  
   getLength()   - 
Method in class selex. SubSeq 
  
   getLength()   - 
Method in class trans.anno. BindingRegion 
 End-Start+1
   getLength()   - 
Method in class trans.anno. Region 
 Returns end - start +1, thus last base is included, not interbase numbering!
   getLength()   - 
Method in class util.bio.annotation. StartStop 
 Assumes interbase coordinates.
   getLength()   - 
Method in class util.bio.digest. Enzyme 
  
   getLENGTH_COMPSEQ()   - 
Method in class gata.aligner. AlignParams 
  
   getLENGTH_REFSEQ()   - 
Method in class gata.aligner. AlignParams 
  
   getLenMax()   - 
Method in class selex. SelexParams 
  
   getLenMin()   - 
Method in class selex. SelexParams 
  
   getLine()   - 
Method in class trans.cel. CelLine 
  
   getLine()   - 
Method in class trans.tpmap. TPMapLine 
  
   getLine()   - 
Method in class util.bio.cluster. Cluster 
  
   getLine()   - 
Method in class util.bio.wrappers. Primer3Pick 
  
   getLineNumber()   - 
Method in class trans.tpmap. TPMapDuplicateFilter 
  
   getLLAdd1PSPM(double, double, double, double)   - 
Method in class meme. MemeMotif 
  
   getLLMemePSPM(double, double, double, double)   - 
Method in class meme. MemeMotif 
  
   getLocalAlignString(int, int)   - 
Method in class gata.aligner. LocalAlignment 
  
   getLocation()   - 
Method in class bioroot.antibody. AntibodyPrep 
  
   getLocation()   - 
Method in class bioroot. ReagentBean 
  
   getLogFile()   - 
Method in class trans.main. T2Parameter 
  
   getLoginName()   - 
Method in class bioroot. LoginBean 
  
   getLoginName()   - 
Method in class bioroot. UserBean 
  
   getLoginName()   - 
Method in class bioroot. UserRegistrationBean 
  
   getLoginName()   - 
Method in class cgar.beans. LoginBean 
  
   getLoginName()   - 
Method in class cgar.beans. UserBean 
  
   getLogTPvalue()   - 
Method in class trans.main. WilcoxonRankSumTest 
  
   getLongitude()   - 
Method in class util.apps. HouseBean 
  
   getLooseMatches()   - 
Method in class bioroot. SearchHit 
  
   getMarkers(DBUtil)   - 
Method in class bioroot.plasmid. PlasmidBean 
  
   getMarkers(DBUtil)   - 
Method in class bioroot.strain. StrainBean 
  
   getMask()   - 
Method in class gata.aligner. AlignerPreferences 
  
   getMaskValue()   - 
Method in class trans.graphics. CelMasker 
  
   getMatch()   - 
Method in class gata.aligner. AlignerPreferences 
  
   getMATCH()   - 
Method in class gata.aligner. AlignParams 
  
   getMatches()   - 
Method in class trans.main. Oligo 
  
   getMatches()   - 
Method in class trans.tpmap. BlastFilterResult 
  
   getMatchId()   - 
Method in class util.bio.wrappers. BlastResult 
  
   getMatching()   - 
Method in class bioroot. SearchBean 
  
   getMatchLength()   - 
Method in class util.bio.wrappers. BlastResult 
  
   getMatchName()   - 
Method in class util.bio.wrappers. BlastResult 
  
   getMatingType()   - 
Method in class bioroot.strain. StrainBean 
  
   getMAX_SCORE()   - 
Method in class gata.aligner. AlignParams 
  
   getMaxBit()   - 
Method in class gata.plotter. ToolsFrame 
  
   getMaxCluster()   - 
Method in class trans.main. Interval 
  
   getMaxE()   - 
Method in class gata.plotter. ToolsFrame 
  
   getMaxGap()   - 
Method in class trans.main. T2Parameter 
  
   getMaxHeight()   - 
Method in class gata.menu. FileInputPanel 
  
   getMaxHeight()   - 
Method in class gata.menu. SaveImagePanel 
  
   getMaximum()   - 
Method in class trans.qc. StatFlag 
  
   getMaximum()   - 
Method in class util.gen. Histogram 
  
   getMaxIntensityValueToPlot()   - 
Method in class trans.graphics. CelMasker 
  
   getMaxMemory()   - 
Method in class trans.main. T2Parameter 
  
   getMaxNumberPeaks()   - 
Method in class trans.main. T2Parameter 
  
   getMaxNumFeatures()   - 
Method in class trans.tpmap. WindowMaker 
  
   getMaxNumPeaks()   - 
Method in class trans.main. PeakPicker 
  
   getMaxWidth()   - 
Method in class gata.menu. FileInputPanel 
  
   getMaxWidth()   - 
Method in class gata.menu. SaveImagePanel 
  
   getMaxWindowFilter()   - 
Method in class edu.utah.seq.data. HeatMapMakerPosNeg 
  
   getMaxWindowFilter()   - 
Method in class trans.main. WindowBlockMakerTwoColor 
  
   getMean()   - 
Method in class trans.qc. CelFileStats 
  
   getMean()   - 
Method in class util.gen. Bin 
  
   getMedian()   - 
Method in class trans.main. ScoredGene 
  
   getMedian()   - 
Method in class trans.qc. StatFlag 
  
   getMedian()   - 
Method in class util.gen. Bin 
  
   getMedianBrightControls()   - 
Method in class trans.qc. CelFileStats 
  
   getMedianDimControls()   - 
Method in class trans.qc. CelFileStats 
  
   getMedianNoSynthControls()   - 
Method in class trans.qc. CelFileStats 
  
   getMedianRatio()   - 
Method in class trans.main. SubWindow 
  
   getMedianScalar()   - 
Method in class trans.qc. CelFileStats 
  
   getMegaCluster()   - 
Method in class util.bio.cluster. HierarchicalClustering 
  
   getMemeLLPSPMCutOffScore()   - 
Method in class meme. MotifSearchResult 
  
   getMemeMotif()   - 
Method in class meme. MotifSearchResult 
  
   getMemeMotifs()   - 
Method in class meme. MemeParser 
  
   getMemeParser()   - 
Method in class meme. MemeMotif 
  
   getMemePSPM()   - 
Method in class meme. MemeMotif 
  
   getMemeReports()   - 
Method in class meme. MemeResults 
  
   getMenu()   - 
Method in class util.apps.gump. Gump 
  
   getMessage()   - 
Method in class bioroot. ContactBean 
  
   getMessage(LabGroup, boolean)   - 
Static method in class bioroot. CronArchive 
  
   getMessage(String)   - 
Static method in class bioroot. Spammer 
  
   getMessages()   - 
Method in class bioroot. CellTypeBean 
  
   getMessages()   - 
Method in class bioroot. ContactBean 
  
   getMessages()   - 
Method in class bioroot. GeneBean 
  
   getMessages()   - 
Method in class bioroot. LoginBean 
  
   getMessages()   - 
Method in class bioroot. MarkerBean 
  
   getMessages()   - 
Method in class bioroot. OrganismBean 
  
   getMessages()   - 
Method in class bioroot. ReagentBean 
  
   getMessages()   - 
Method in class bioroot. SearchBean 
  
   getMessages()   - 
Method in class bioroot. UserRegistrationBean 
  
   getMessages()   - 
Method in class cgar.beans. LoginBean 
  
   getMethSens()   - 
Method in class util.bio.digest. Enzyme 
  
   getMiddle()   - 
Method in class trans.roc. RocWindow 
  
   getMiddle()   - 
Method in class util.gen. Bin 
  
   getMIN_SCORE()   - 
Method in class gata.aligner. AlignParams 
  
   getMinBit()   - 
Method in class gata.plotter. ToolsFrame 
  
   getMinBpSides()   - 
Method in class trans.main. PeakPicker 
  
   getMinE()   - 
Method in class gata.plotter. ToolsFrame 
  
   getMinimalCorrelationCoefficient()   - 
Method in class util.bio.cluster. HierarchicalClustering 
  
   getMinimum()   - 
Method in class trans.qc. StatFlag 
  
   getMinimum()   - 
Method in class util.gen. Histogram 
  
   getMinimumNumberOfOligos()   - 
Method in class trans.main. T2Parameter 
  
   getMinLength()   - 
Method in class util.bio.parsers. MultiFastaParser 
  
   getMinNumberOligosInSubWin()   - 
Method in class trans.main. T2Parameter 
  
   getMinPercent()   - 
Method in class trans.main. PeakPicker 
  
   getMinRun()   - 
Method in class trans.main. PeakPicker 
  
   getMinWindowFilter()   - 
Method in class edu.utah.seq.data. HeatMapMakerPosNeg 
  
   getMinWindowFilter()   - 
Method in class trans.main. WindowBlockMakerTwoColor 
  
   getMisc()   - 
Method in class trans.main. ScoredGene 
  
   getMisMatch()   - 
Method in class gata.aligner. AlignerPreferences 
  
   getMISMATCH()   - 
Method in class gata.aligner. AlignParams 
  
   getMls()   - 
Method in class util.apps. HouseBean 
  
   getMMX()   - 
Method in class trans.tpmap. TPMapFeature 
  
   getMMY()   - 
Method in class trans.tpmap. TPMapFeature 
  
   getModel()   - 
Method in class util.gen. LinearRegression 
 Returns Y=mX+b with full precision, no rounding of numbers.
   getModelPoints()   - 
Method in class trans.cel. SplineScalar 
  
   getModifications()   - 
Method in class bioroot.oligo. OligoBean 
  
   getMotifDesc()   - 
Method in class meme. MemeMotif 
  
   getMotifHitPlot()   - 
Method in class meme. MemeResults 
 Rude and crude method to plot the percent hits of each motif to each file using data from motifHitSummary
   getMotifHitPlotText()   - 
Method in class meme. MemeResults 
  
   getMotifHits()   - 
Method in class meme. MotifScanner 
  
   getMotifHitsSummary()   - 
Method in class meme. MemeResults 
 Method to return a summary of all the motif hits to each file, modify this to build an HTML table
   getMotifHitsSummaryText()   - 
Method in class meme. MemeResults 
  
   getMotifHitSum()   - 
Method in class trans.main. Interval 
 Sums the logged motif hit values using the best AID window or the entire interval.
   getMotifSig()   - 
Method in class meme. MotifSearchResult 
  
   getMotifSites()   - 
Method in class meme. MemeMotif 
  
   getMotifSumLn()   - 
Method in class meme. MemeMotif 
  
   getMotifSummary()   - 
Method in class meme. MemeResults 
 Method to return a summary of all the motifs generated by MemeR, modify this to build an HTML table
   getMotifSummaryText()   - 
Method in class meme. MemeResults 
  
   getMutagen()   - 
Method in class bioroot.strain. StrainBean 
  
   getMW(String)   - 
Method in class bioroot.oligo. OligoBean 
  
   getN()   - 
Method in class trans.main. WilcoxonSignedRankTest 
  
   getN()   - 
Method in class util.bio.parsers. BaseCount 
  
   getName()   - 
Method in class bioroot.antibody. AntibodyPrep 
  
   getName()   - 
Method in class bioroot. BlastMatcher 
  
   getName()   - 
Method in class bioroot. CellTypeBean 
  
   getName()   - 
Method in class bioroot. Feature 
  
   getName()   - 
Method in class bioroot. GeneBean 
  
   getName()   - 
Method in class bioroot. IdNameMatch 
  
   getName()   - 
Method in class bioroot. LabGroup 
  
   getName()   - 
Method in class bioroot. MarkerBean 
  
   getName()   - 
Method in class bioroot. OrganismBean 
  
   getName()   - 
Method in class bioroot. Organization 
  
   getName()   - 
Method in class bioroot.plasmid. OligoMatch 
  
   getName()   - 
Method in class bioroot. ReagentBean 
  
   getName()   - 
Method in class bioroot. TableHeading 
  
   getName()   - 
Method in class cgar.beans. AnalysisBean 
  
   getName()   - 
Method in class cgar.beans. AnalysisGroupBean 
  
   getName()   - 
Method in class cgar.beans. AnalysisTypeBean 
  
   getName()   - 
Method in class cgar.beans. GenomeBean 
  
   getName()   - 
Method in class cgar.beans. LabGroupBean 
  
   getName()   - 
Method in class cgar.beans. ProcessedDataBean 
  
   getName()   - 
Method in class cgar.beans. ProcessedDataTypeBean 
  
   getName()   - 
Method in class cgar.beans. RawDataBean 
  
   getName()   - 
Method in class cgar.beans. SpeciesBean 
  
   getName()   - 
Method in class cgar.beans. UserTypeBean 
  
   getName()   - 
Method in class cgar.beans. VisibilityBean 
  
   getName()   - 
Method in class edu.utah.seq.data. Info 
  
   getName()   - 
Method in class edu.utah.seq.methylation454. Reference 
  
   getName()   - 
Method in class expr. ExpressedGene 
  
   getName()   - 
Method in class expr. MiRNA 
  
   getName()   - 
Method in class expr. MiRNATarget 
  
   getName()   - 
Method in class trans.main. PosRegion 
  
   getName()   - 
Method in class trans.qc. StatFlag 
  
   getName()   - 
Method in class trans.roc. ControlGene 
  
   getName()   - 
Method in class util.bio.annotation. ExonIntron 
  
   getName()   - 
Method in class util.bio.annotation. GeneGroup 
  
   getName()   - 
Method in class util.bio.annotation. GenericFeature 
  
   getName()   - 
Method in class util.bio.annotation. Transcript 
  
   getName()   - 
Method in class util.bio.cluster. Cluster 
  
   getName()   - 
Method in class util.bio.digest. Enzyme 
  
   getName()   - 
Method in class util.bio.parsers. Fasta 
  
   getName()   - 
Method in class util.bio.parsers.gff. Gff3Feature 
 Reserved Attribute.
   getName()   - 
Method in class util.bio.parsers. UCSCGeneLine 
  
   getName()   - 
Method in class util.gen. FileSize 
  
   getNAME_COMPSEQ()   - 
Method in class gata.aligner. AlignParams 
  
   getNAME_REFSEQ()   - 
Method in class gata.aligner. AlignParams 
  
   getNameLabel()   - 
Method in class util.bio.cluster. Cluster 
  
   getNameLabelCenterYCoordinate()   - 
Method in class util.bio.cluster. Cluster 
  
   getNameLowerCase()   - 
Method in class bioroot. IdNameMatch 
  
   getNames()   - 
Method in class expr. CorrelationWindow 
  
   getNames()   - 
Method in class trans.cel. ControlStats 
  
   getNames()   - 
Method in class util.bio.parsers. MultiFastaParser 
  
   getNames()   - 
Method in class util.bio.parsers. QualityFileParser 
  
   getNamesAL()   - 
Method in class util.bio.parsers. MultiFastaParser 
  
   getNamesSeqs()   - 
Method in class util.bio.parsers. MultiFastaParser 
 Returns a hashmap of the fasta name: sequence
   getNearestNbrTm(String)   - 
Method in class bioroot.oligo. OligoBean 
 Returns -1 if non GATC's are found.
   getNeighborhood()   - 
Method in class trans.anno. BindingRegion 
  
   getNeighboringGeneGrps()   - 
Method in class trans.anno. BindingRegion 
  
   getNgUl()   - 
Method in class bioroot.oligo. OligoBean 
  
   getNonAmbiguousLetters()   - 
Static method in class util.gen. Passwords 
  
   getNonCodingSegments()   - 
Method in class util.bio.annotation. GeneRep 
 Conservative estimate of non coding segments between coding exons.
   getNonOverlapYCoor()   - 
Method in class gata.geneGlyphs. GeneRepGlyph 
  
   getNormalDNALines()   - 
Method in class gata.main. AnnoSpecParams 
  
   getNormalizedControlReplicas()   - 
Method in class trans.main. T2Parameter 
  
   getNormalizedTreatmentReplicas()   - 
Method in class trans.main. T2Parameter 
  
   getNormalStroke()   - 
Method in class gata.main. GATAParams 
  
   getNote()   - 
Method in class util.bio.parsers.gff. Gff3Feature 
 Reserved Attribute.
   getNotes()   - 
Method in class bioroot.antibody. AntibodyPrep 
  
   getNotes()   - 
Method in class bioroot.antibody. UseDilution 
  
   getNotes()   - 
Method in class bioroot. CellTypeBean 
  
   getNotes()   - 
Method in class bioroot. GeneBean 
  
   getNotes()   - 
Method in class bioroot. MarkerBean 
  
   getNotes()   - 
Method in class bioroot.oligo. OligoUploadBean 
  
   getNotes()   - 
Method in class bioroot. OrganismBean 
  
   getNotes()   - 
Method in class bioroot.plasmid. PlasmidUploadBean 
  
   getNotes()   - 
Method in class bioroot. ReagentBean 
  
   getNotes()   - 
Method in class bioroot.strain. StrainUploadBean 
  
   getNotes()   - 
Method in class cgar.beans. GenomeBean 
  
   getNotes()   - 
Method in class edu.utah.seq.data. Info 
  
   getNotes()   - 
Method in class trans.anno. Region 
  
   getNotes()   - 
Method in class trans.graphics. GenomicRegion 
  
   getNotes()   - 
Method in class trans.qc. CelFileStats 
  
   getNotes()   - 
Method in class trans.roc. ControlGene 
  
   getNotes()   - 
Method in class util.apps.gump. Option 
  
   getNotes()   - 
Method in class util.bio.parsers. UCSCGeneLine 
  
   getNtAtXZero()   - 
Method in class gata.main. AnnoSpecParams 
  
   getNtPerPixel()   - 
Method in class gata.main. GATAParams 
  
   getNumber()   - 
Method in class util.bio.parsers. Fasta 
  
   getNumberChromosome()   - 
Static method in class trans.misc. Util 
 Creates a String[] containing [1]"chr1", [2]"chr2" ...
   getNumberControlIntensities()   - 
Method in class trans.main. Interval 
  
   getNumberCs()   - 
Method in class edu.utah.seq.methylation454. CpG 
  
   getNumberLetters()   - 
Static method in class util.gen. Passwords 
  
   getNumberMisMatches()   - 
Method in class trans.bkgrnd. LoadedOligo 
  
   getNumberMotifHits()   - 
Method in class trans.main. Interval 
  
   getNumberName()   - 
Method in class meme. MemeMotif 
  
   getNumberObservations()   - 
Method in class edu.utah.seq.data. PointData 
  
   getNumberOfBins()   - 
Method in class util.gen. Histogram 
  
   getNumberOfCuts()   - 
Method in class util.bio.digest. Enzyme 
  
   getNumberOfIntervals()   - 
Method in class trans.main. T2Parameter 
  
   getNumberOfRandomPermutations()   - 
Method in class trans.main. T2Parameter 
  
   getNumberOfWindows()   - 
Method in class edu.utah.seq.data. EnrichedRegion 
  
   getNumberOfWindows()   - 
Method in class trans.main. Interval 
  
   getNumberOligos()   - 
Method in class trans.main. ChromSet 
  
   getNumberOligos()   - 
Method in class trans.main. Window 
  
   getNumberPositionValues()   - 
Method in class edu.utah.seq.parsers. BarParser 
  
   getNumberPrepUseLines()   - 
Method in class bioroot.antibody. AntibodyBean 
  
   getNumberReads()   - 
Method in class edu.utah.seq.methylation454. CpG 
  
   getNumberToDisplay()   - 
Method in class bioroot. Preference 
  
   getNumberTreatmentIntensities()   - 
Method in class trans.main. Interval 
  
   getNumberTreatmentsControls()   - 
Method in class trans.main. WilcoxonRankSumTest 
  
   getNumberWMinusRanks()   - 
Method in class trans.main. WilcoxonSignedRankTest 
  
   getNumberWPlusRanks()   - 
Method in class trans.main. WilcoxonSignedRankTest 
  
   getNumBrightOutliers()   - 
Method in class trans.qc. CelFileStats 
  
   getNumDimOutliers()   - 
Method in class trans.qc. CelFileStats 
  
   getNumHitsAddOneLLPSPM()   - 
Method in class meme. MotifSearchResult 
  
   getNumHitsMemeLLPSPM()   - 
Method in class meme. MotifSearchResult 
  
   getNumLessThanMin()   - 
Method in class util.gen. Histogram 
  
   getNumMoreThanMax()   - 
Method in class util.gen. Histogram 
  
   getNumMotifs()   - 
Method in class meme. MemeParser 
  
   getNumNegatives()   - 
Method in class trans.roc. Bin 
  
   getNumNoSynthOutliers()   - 
Method in class trans.qc. CelFileStats 
  
   getNumOligos()   - 
Method in class trans.main. PeakPicker 
  
   getNumPermutations()   - 
Method in class trans.main. ScanChromosomes 
  
   getNumPermutations()   - 
Method in class trans.main. ScanChromosomesCNV 
  
   getNumPositives()   - 
Method in class trans.roc. Bin 
  
   getNumReads()   - 
Method in class selex. SeqFiles 
  
   getNumReads()   - 
Method in class util.bio.parsers. MultiFastaParser 
  
   getnumSeqsSearched()   - 
Method in class meme. MotifSearchResult 
  
   getNumStretchesGoodQuality(int[], int, int)   - 
Static method in class selex. SeqRead 
 Counts the number of stretches of scores above a cut off in an int[].
   getObjectsFile()   - 
Method in class gata.main. GATAParams 
  
   getObjectsFile()   - 
Method in class gata.main. GATAPlotter 
  
   getOldFileName()   - 
Method in class bioroot.antibody. AntibodyBean 
  
   getOldFileName()   - 
Method in class bioroot.antibody. AntibodyPrep 
  
   getOligoBeans()   - 
Method in class bioroot.oligo. OligoUploadBean 
  
   getOligoForm(OligoBean, DBUtil, UserBean)   - 
Static method in class bioroot.oligo. OligoBase 
 Returns an oligo form filled it with the values in the oligo bean.
   getOligoMatches()   - 
Method in class bioroot.plasmid. PlasmidBean 
  
   getOligoPreference()   - 
Method in class bioroot. UserBean 
  
   getOligoPrefs()   - 
Method in class bioroot. UserBean 
  
   getOligoReport(OligoBean, DBUtil, UserBean, boolean)   - 
Static method in class bioroot.oligo. OligoBase 
 Returns an oligo report.
   getOligoReports()   - 
Method in class bioroot.oligo. OligoUploadBean 
  
   getOligos()   - 
Method in class trans.main. Interval 
  
   getOligos()   - 
Method in class trans.main. PeakPicker 
  
   getOligos()   - 
Method in class trans.main. SubWindow 
  
   getOneBPMisMatches()   - 
Method in class trans.tpmap. BlastFilterResult 
  
   getOntologyTerm()   - 
Method in class util.bio.parsers.gff. Gff3Feature 
 Reserved Attribute.
   getOptions()   - 
Method in class util.apps.gump. Gump 
  
   getOrder()   - 
Method in class bioroot. Feature 
  
   getOrderBy()   - 
Method in class bioroot. Preference 
  
   getOrderBy()   - 
Method in class bioroot. ReagentBean 
  
   getOrderNumber()   - 
Method in class bioroot. TableHeading 
  
   getOrganismId()   - 
Method in class bioroot.antibody. AntibodyPrep 
  
   getOrganismId(String)   - 
Method in class bioroot. DBUtil 
 Fetches an organismId given a organismName, assumes connection to BioRoot.
   getOrganismId()   - 
Method in class bioroot. ReagentBean 
  
   getOrganismName(DBUtil)   - 
Method in class bioroot.antibody. AntibodyPrep 
  
   getOrganismName(int)   - 
Method in class bioroot. DBUtil 
 Fetches an organismName given an organismId, assumes connection to BioRoot.
   getOrganismName(DBUtil)   - 
Method in class bioroot. ReagentBean 
  
   getOrganization(DBUtil)   - 
Method in class bioroot. ReagentBean 
  
   getOrganization()   - 
Method in class bioroot. UserBean 
  
   getOri()   - 
Method in class gata.aligner. LocalAlignment 
  
   getOrientation()   - 
Method in class bioroot.oligo. OligoBean 
  
   getOrientation()   - 
Method in class gata.aligner. Alignment 
  
   getOrientation()   - 
Method in class meme. MotifHit 
  
   getOrientation()   - 
Method in class util.bio.annotation. GeneGroup 
  
   getOrientation()   - 
Method in class util.bio.annotation. GenericFeature 
  
   getOrientation()   - 
Method in class util.bio.annotation. Transcript 
  
   getOrientation()   - 
Method in class util.bio.annotation. Translation 
  
   getOrientation()   - 
Method in class util.bio.wrappers. BL2SeqHit 
  
   getOriginalOrderNumber()   - 
Method in class bioroot. TableHeading 
  
   getOverHangs()   - 
Method in class util.bio.digest. Enzyme 
  
   getOverlap3PrimeGeneGrps()   - 
Method in class trans.anno. BindingRegion 
  
   getOverlap5PrimeGeneGrps()   - 
Method in class trans.anno. BindingRegion 
  
   getOverlapping()   - 
Method in class trans.main. PosRegion 
  
   getOwnerId()   - 
Method in class bioroot. MarkerBean 
  
   getOwnerId()   - 
Method in class bioroot. OrganismBean 
  
   getOwnerId()   - 
Method in class bioroot. ReagentBean 
  
   getOwnerName()   - 
Method in class bioroot. ContactBean 
  
   getOwnerName(DBUtil)   - 
Method in class bioroot. ReagentBean 
  
   getPanel()   - 
Method in class gata.aligner. AlignerInputForm 
  
   getPanel()   - 
Method in class trans.graphics. IntervalDrawFrame 
  
   getPanelHeight()   - 
Method in class trans.graphics. RankedSetDrawPanel 
  
   getPanelHeight()   - 
Method in class util.bio.cluster. ClusterDrawPanel 
  
   getPanelWidth()   - 
Method in class trans.graphics. RankedSetDrawPanel 
  
   getPanelWidth()   - 
Method in class util.bio.cluster. ClusterDrawPanel 
  
   getParent()   - 
Method in class util.bio.annotation. ExonIntron 
  
   getParent()   - 
Method in class util.bio.parsers.gff. Gff3Feature 
 Reserved Attribute.
   getParentCluster()   - 
Method in class util.bio.cluster. Cluster 
  
   getParentConcats(DBUtil)   - 
Method in class bioroot.strain. StrainBean 
 Returns an array of id: strainName, or empty String[]{""} if there are none.
   getParentLocAlignString()   - 
Method in class gata.aligner. Alignment 
  
   getParentPoint()   - 
Method in class util.bio.cluster. Cluster 
  
   getParsedFileName()   - 
Method in class meme. MemeParser 
  
   getPassageNumber()   - 
Method in class bioroot.strain. StrainBean 
  
   getPassword()   - 
Method in class bioroot. LoginBean 
  
   getPassword()   - 
Method in class bioroot. UserBean 
  
   getPassword()   - 
Method in class cgar.beans. LoginBean 
  
   getPassword1()   - 
Method in class bioroot. UserRegistrationBean 
  
   getPassword2()   - 
Method in class bioroot. UserRegistrationBean 
  
   getPath()   - 
Method in class gata.geneGlyphs. GenericGlyph 
  
   getPathToResults()   - 
Method in class gata.aligner. AlignParams 
  
   getPeakBP()   - 
Method in class trans.main. BindingPeak 
  
   getPeakBp()   - 
Method in class trans.main. PeakPicker 
  
   getPeakIndex()   - 
Method in class trans.main. PeakPicker 
  
   getPeakPickerWindowSize()   - 
Method in class trans.main. T2Parameter 
  
   getPercentFinishedBasement()   - 
Method in class util.apps. HouseBean 
  
   getPermMeanDiff()   - 
Method in class trans.main. SamChromosomeData 
  
   getPermTStats()   - 
Method in class trans.main. SamChromosomeData 
  
   getPermutationType()   - 
Method in class trans.main. T2Parameter 
  
   getPermVariance()   - 
Method in class trans.main. SamChromosomeData 
  
   getPhase()   - 
Method in class util.bio.parsers.gff. Gff3Feature 
  
   getPhenotype()   - 
Method in class bioroot.strain. StrainBean 
  
   getPhone()   - 
Method in class bioroot. UserBean 
  
   getPhone()   - 
Method in class bioroot. UserRegistrationBean 
  
   getPixBtwDNATracks()   - 
Method in class gata.main. GATAParams 
  
   getPixBtwLabelTrack()   - 
Method in class gata.main. GATAParams 
  
   getPixBtwProtRNA()   - 
Method in class gata.main. GATAParams 
  
   getPixBtwTracks()   - 
Method in class gata.main. GATAParams 
  
   getPixBtwTransGrpLabel()   - 
Method in class gata.main. GATAParams 
  
   getPixBtwTransGrps()   - 
Method in class gata.main. GATAParams 
  
   getPixBtwTransGrpsGene()   - 
Method in class gata.main. GATAParams 
  
   getPixelsPerBase()   - 
Method in class trans.graphics. Graph 
  
   getPixFatLineThickness()   - 
Method in class gata.main. GATAParams 
  
   getPixHeightArrows()   - 
Method in class gata.main. GATAParams 
  
   getPixNormalLineThickness()   - 
Method in class gata.main. GATAParams 
  
   getPixPerNt()   - 
Method in class gata.main. GATAParams 
  
   getPixThinLineThickness()   - 
Method in class gata.main. GATAParams 
  
   getPlainTextOligos()   - 
Method in class bioroot.plasmid. PlasmidBean 
 Returns plain text of oligoMatches for archive.
   getPlainTextParent()   - 
Method in class bioroot.strain. StrainBean 
 Returns plain text of parent ids for archive.
   getPlainTextPlasmid()   - 
Method in class bioroot.antibody. AntibodyBean 
 Returns plain text of plasmid ids for archive.
   getPlainTextPlasmid()   - 
Method in class bioroot.strain. StrainBean 
 Returns plain text of plasmid ids for archive.
   getPlainTextStrain()   - 
Method in class bioroot.antibody. AntibodyBean 
 Returns plain text of strain ids for archive.
   getPlainTextStrain()   - 
Method in class bioroot.plasmid. PlasmidBean 
 Returns plain text of strain ids for archive.
   getPlasmidBeans()   - 
Method in class bioroot.plasmid. PlasmidUploadBean 
  
   getPlasmidConcats(DBUtil)   - 
Method in class bioroot.antibody. AntibodyBean 
 Returns an array of id: plasmidName, or empty String[]{""} if there are none.
   getPlasmidConcats(DBUtil)   - 
Method in class bioroot.strain. StrainBean 
 Returns an array of id: plasmidName, or empty String[]{""} if there are none.
   getPlasmidForm(PlasmidBean, DBUtil, UserBean)   - 
Static method in class bioroot.plasmid. PlasmidBase 
 Returns a plasmid form filled with the values in the  bean.
   getPlasmidPreference()   - 
Method in class bioroot. UserBean 
  
   getPlasmidPrefs()   - 
Method in class bioroot. UserBean 
  
   getPlasmidReport(PlasmidBean, DBUtil, UserBean, boolean)   - 
Static method in class bioroot.plasmid. PlasmidBase 
 Returns an plasmid report.
   getPlasmidReports()   - 
Method in class bioroot.plasmid. PlasmidUploadBean 
  
   getPlotterPreferences()   - 
Method in class gata.main. GATAParams 
  
   getPMX()   - 
Method in class trans.tpmap. TPMapFeature 
  
   getPmX()   - 
Method in class trans.tpmap. TPMapLine 
  
   getPMY()   - 
Method in class trans.tpmap. TPMapFeature 
  
   getPmY()   - 
Method in class trans.tpmap. TPMapLine 
  
   getPngResultFile()   - 
Method in class util.bio.cluster. HierarchicalClustering 
  
   getPoint()   - 
Method in class trans.graphics. GenomicRegionGlyph 
  
   getPosition()   - 
Method in class edu.utah.seq.data. Point 
  
   getPosition()   - 
Method in class edu.utah.seq.parsers. ElandExportLine 
  
   getPosition()   - 
Method in class trans.roc. Gr 
  
   getPosition()   - 
Method in class trans.roc. Sgr 
  
   getPosition()   - 
Method in class trans.tpmap. TPMapFeature 
  
   getPosition()   - 
Method in class util.bio.parsers. BaseCount 
  
   getPositions()   - 
Method in class edu.utah.seq.data. PointData 
 Will load positions and scores from file if null.
   getPositions()   - 
Method in class trans.graphics. Graph 
  
   getPositions()   - 
Method in class trans.main. ScanChromosomes 
  
   getPositions()   - 
Method in class trans.main. ScanChromosomesCNV 
  
   getPrice()   - 
Method in class util.apps. HouseBean 
  
   getPrintWriter()   - 
Method in class util.apps. FileSplitter 
  
   getProcessedDataTypeId()   - 
Method in class cgar.beans. ProcessedDataBean 
  
   getProteinColor()   - 
Method in class gata.main. GATAParams 
  
   getProtocolId()   - 
Method in class cgar.beans. AnalysisBean 
  
   getProtocolNotes()   - 
Method in class cgar.beans. AnalysisBean 
  
   getPurification()   - 
Method in class bioroot.antibody. AntibodyPrep 
  
   getPurification()   - 
Method in class bioroot.oligo. OligoBean 
  
   getPurification()   - 
Method in class bioroot.plasmid. PlasmidBean 
  
   getPvalue()   - 
Method in class expr. CorrelationWindow 
  
   getPvalue()   - 
Method in class expr. MiRNATarget 
  
   getPValue()   - 
Method in class trans.main. WilcoxonSignedRankTest 
 A p-value of -1 is the default insignificant number.
   getQualFile(int)   - 
Method in class selex. SelexParams 
  
   getQualFile()   - 
Method in class selex. SeqFiles 
  
   getQualFiles()   - 
Method in class selex. SelexParams 
  
   getQualNames()   - 
Method in class util.bio.parsers. QualityFileParser 
 Returns a hashmap of the fasta name: sequence
   getQuals()   - 
Method in class util.bio.parsers. QualityFileParser 
  
   getQualScores()   - 
Method in class selex. SubSeq 
  
   getQuantiles()   - 
Method in class trans.cel. QuantileNormalization 
 Fetches an array of sorted normalized Quantiles
   getQuartiles()   - 
Method in class trans.qc. CelFileStats 
  
   getRank()   - 
Method in class trans.anno. BindingRegion 
  
   getRank()   - 
Method in class trans.graphics. GenomicRegion 
  
   getRank()   - 
Method in class trans.main. WilcoxonSamplePair 
  
   getRanks()   - 
Method in class util.gen. RankedFloatArray 
  
   getRApp()   - 
Method in class trans.main. T2Parameter 
  
   getRatio()   - 
Method in class trans.misc. GCRatio 
  
   getRawMedian()   - 
Method in class trans.qc. CelFileStats 
  
   getRawScore()   - 
Method in class util.bio.wrappers. BL2SeqHit 
  
   getRawScoreCutOff()   - 
Method in class util.bio.wrappers. BL2Seq 
  
   getRawUnparsedResults()   - 
Method in class util.bio.wrappers. BL2Seq 
  
   getRead()   - 
Method in class edu.utah.seq.methylation454. DataLine 
  
   getReadLength()   - 
Method in class edu.utah.seq.data. Info 
  
   getReads()   - 
Method in class edu.utah.seq.methylation454. CpG 
  
   getReagentNames()   - 
Method in class bioroot. ContactBean 
  
   getRealMeanDiff()   - 
Method in class trans.main. SamChromosomeData 
  
   getRealTStats()   - 
Method in class trans.main. SamChromosomeData 
  
   getRealVariance()   - 
Method in class trans.main. SamChromosomeData 
  
   getRecipients()   - 
Method in class bioroot.antibody. AntibodyPrep 
  
   getRecipients()   - 
Method in class bioroot.plasmid. PlasmidBean 
  
   getRecipients()   - 
Method in class bioroot.strain. StrainBean 
  
   getRecogSeq()   - 
Method in class util.bio.digest. Enzyme 
  
   getRectangle()   - 
Method in class trans.graphics. GenomicRegionGlyph 
  
   getRefAnnoSpecParams()   - 
Method in class gata.main. GATAParams 
  
   getReference()   - 
Method in class bioroot. ReagentBean 
  
   getReferenceY()   - 
Method in class gata.main. AnnoSpecParams 
  
   getRefSeq()   - 
Method in class gata.aligner. AlignerPreferences 
  
   getRefSeqFile()   - 
Method in class gata.aligner. AlignParams 
  
   getRefStart()   - 
Method in class gata.aligner. AlignerPreferences 
  
   getRefTexF()   - 
Method in class gata.aligner. AlignerInputPanel 
  
   getRegEx()   - 
Method in class util.bio.digest. Enzyme 
  
   getRegExGeneTypes()   - 
Method in class util.bio.parsers.gff. DmelRel4Extractor 
  
   getRegExTypes()   - 
Method in class util.bio.parsers.gff. Gff3Parser 
  
   getRegion()   - 
Method in class trans.main. PosRegion 
  
   getRegionNames()   - 
Method in class trans.main. Interval 
  
   getRegionsOne()   - 
Method in class trans.graphics. RankedSetAnalysis 
  
   getRegionsTwo()   - 
Method in class trans.graphics. RankedSetAnalysis 
  
   getRelativeCoordinates()   - 
Method in class gata.aligner. Alignment 
  
   getRemainingAliquots()   - 
Method in class bioroot.antibody. AntibodyPrep 
  
   getRemainingAliquots()   - 
Method in class bioroot.strain. StrainBean 
  
   getRemoteHost()   - 
Method in class bioroot. UserBean 
  
   getRepeatRegionFiles()   - 
Method in class trans.main. T2Parameter 
  
   getRequesterEmail()   - 
Method in class bioroot. ContactBean 
  
   getResponse()   - 
Method in class bioroot. LoginBean 
  
   getRestock()   - 
Method in class bioroot.strain. StrainBean 
  
   getRestrictionCutSites(Enzyme[])   - 
Method in class bioroot.oligo. OligoBean 
  
   getRestrictionCutSites(Enzyme[])   - 
Method in class bioroot.plasmid. PlasmidBean 
  
   getRestrictionMapString(String, Enzyme[])   - 
Static method in class util.bio.digest. REDigest 
  
   getRestSite()   - 
Method in class selex. SelexParams 
  
   getResult(ResultSet)   - 
Static method in class bioroot. DBUtil 
  
   getResults()   - 
Method in class meme. MemeResults 
  
   getResults()   - 
Method in class util.gen. SQL 
  
   getResultsDirectory()   - 
Method in class trans.main. ScanChromosomes 
  
   getResultsDirectory()   - 
Method in class trans.main. ScanChromosomesCNV 
  
   getResultsDirectory()   - 
Method in class trans.main. T2Parameter 
  
   getResultSetInt(String)   - 
Method in class bioroot. DBUtil 
 Use to avoid a null return when attempting to fetch a ResultSet.getInt().
   getResultSetInt(String)   - 
Method in class util.gen. SQL 
 Use to avoid a null return when attempting to fetch a ResultSet.getInt().
   getResultSetString(String)   - 
Method in class bioroot. DBUtil 
 Use to avoid a null return when attempting to fetch a ResultSet.getString().
   getResultSetString(String)   - 
Method in class util.gen. SQL 
 Use to avoid a null return when attempting to fetch a ResultSet.getString().
   getResultsName()   - 
Method in class trans.main. T2Parameter 
  
   getResultString()   - 
Method in class bioroot. DBUtil 
 returns the first column values from a result, for testing
   getResultSummaryLine()   - 
Method in class util.bio.wrappers. Primer3Pick 
  
   getRevCompRef()   - 
Method in class util.bio.xamer. Xamer 
  
   getReversedBinCounts()   - 
Method in class util.gen. Histogram 
  
   getRfa()   - 
Method in class expr. ExpressedGene 
  
   getRightFlankingIndex()   - 
Method in class trans.main. BindingPeak 
  
   getRightIndex()   - 
Method in class trans.main. PeakPicker 
  
   getRightLR()   - 
Method in class trans.cel. SplineScalar 
  
   getRightSideMatch()   - 
Method in class selex. SelexParams 
  
   getRightSideReplace()   - 
Method in class selex. SelexParams 
  
   getRightSlope()   - 
Method in class trans.main. Oligo 
  
   getRNAColor()   - 
Method in class gata.main. GATAParams 
  
   getRoundedModel()   - 
Method in class util.gen. LinearRegression 
 Returns Y=mX+b
   getRowsEffected()   - 
Method in class bioroot. DBUtil 
  
   getRSquared()   - 
Method in class util.gen. LinearRegression 
  
   getRunning()   - 
Method in class selex. SelexParams 
  
   getRunningX()   - 
Method in class trans.graphics. IntervalDrawPanel 
  
   getRunningY()   - 
Method in class trans.graphics. IntervalDrawPanel 
  
   getS3Service()   - 
Method in class util.amazon. S3Manager 
  
   getSample()   - 
Method in class edu.utah.seq.methylation454. DataLine 
  
   getSamples()   - 
Method in class edu.utah.seq.methylation454. MethylationParser 
  
   getSaveDirectory()   - 
Method in class trans.main. ChromSet 
  
   getScaleBar()   - 
Method in class gata.main. AnnoSpecParams 
  
   getScaleBarLabel()   - 
Method in class gata.geneGlyphs. ScaleBar 
  
   getScaleBarLines()   - 
Method in class gata.geneGlyphs. ScaleBar 
  
   getScaleBarWidth()   - 
Method in class gata.geneGlyphs. ScaleBar 
  
   getScaleBarX()   - 
Method in class gata.main. AnnoSpecParams 
  
   getScaleBarY()   - 
Method in class gata.main. AnnoSpecParams 
  
   getScaleLineColor()   - 
Method in class gata.main. AnnoSpecParams 
  
   getScaleLines()   - 
Method in class gata.geneGlyphs. ScaleBar 
  
   getScaleRulerLines()   - 
Method in class gata.geneGlyphs. ScaleBar 
  
   getScaleRulerY()   - 
Method in class gata.main. AnnoSpecParams 
  
   getSchedule()   - 
Method in class bioroot. LabGroup 
  
   getScore()   - 
Method in class bioroot. SearchHit 
  
   getScore()   - 
Method in class edu.utah.seq.data. Point 
  
   getScore()   - 
Method in class edu.utah.seq.parsers. ElandExportLine 
  
   getScore()   - 
Method in class expr. CorrelationWindow 
  
   getScore()   - 
Method in class expr. MiRNATarget 
  
   getScore()   - 
Method in class gata.aligner. AlignerPreferences 
  
   getScore()   - 
Method in class gata.aligner. Alignment 
  
   getScore()   - 
Method in class gata.geneGlyphs. GenericGlyph 
  
   getScore()   - 
Method in class meme. MotifHit 
  
   getScore()   - 
Method in class trans.anno. BindingRegion 
  
   getScore()   - 
Method in class trans.graphics. GenomicRegion 
  
   getScore()   - 
Method in class trans.main. BindingPeak 
  
   getScore()   - 
Method in class trans.roc. Gr 
  
   getScore()   - 
Method in class trans.roc. RocWindow 
  
   getScore()   - 
Method in class trans.roc. Sgr 
  
   getScore()   - 
Method in class util.bio.annotation. GenericFeature 
  
   getScore()   - 
Method in class util.bio.annotation. ScoredCoordinate 
  
   getScore()   - 
Method in class util.bio.parsers.gff. Gff3Feature 
  
   getScore()   - 
Method in class util.bio.parsers.gff. GffFeature 
  
   getScore()   - 
Method in class util.bio.wrappers. BlastResult 
  
   getSCORE_DESCRIPTION()   - 
Static method in class trans.main. ScanChip 
  
   getSCORE_DESCRIPTION()   - 
Static method in class trans.main. ScanChromosomes 
  
   getSCORE_DESCRIPTION()   - 
Static method in class trans.main. ScanChromosomesCNV 
  
   getScoreCutOff()   - 
Method in class selex. SelexParams 
  
   getScoreCutOff()   - 
Method in class trans.main. PeakPicker 
  
   getScoreIndex()   - 
Method in class edu.utah.seq.data. HeatMapMaker 
  
   getScoreIndex()   - 
Method in class trans.main. WindowBlockMaker 
  
   getScores()   - 
Method in class edu.utah.seq.data. PointData 
 Will load scores and positions from file if null.
   getScores()   - 
Method in class edu.utah.seq.data. SmoothingWindow 
  
   getScores()   - 
Method in class trans.main. Window 
  
   getScores()   - 
Method in class trans.roc. Positive 
  
   getScores()   - 
Method in class util.bio.parsers. UCSCGeneLine 
  
   getScrollPane()   - 
Method in class trans.graphics. VirtualCelFrame 
  
   getSearchColumnNames()   - 
Static method in class bioroot.antibody. AntibodyBase 
 Returns a list of the search column names used in an SQL statment , ie Oligo.name, 1/2 the searchHash.
   getSearchColumnNames()   - 
Static method in class bioroot.oligo. OligoBase 
 Returns a list of the search column names used in an SQL statment , ie Oligo.name, 1/2 the searchHash.
   getSearchColumnNames()   - 
Static method in class bioroot.plasmid. PlasmidBase 
 Returns a list of the search column names used in an SQL statment , ie Oligo.name, 1/2 the searchHash.
   getSearchColumnNames()   - 
Static method in class bioroot.strain. StrainBase 
 Returns a list of the search column names used in an SQL statment , ie name, 1/2 the searchHash.
   getSearchedSeqFile()   - 
Method in class meme. MotifSearchResult 
  
   getSearchFor()   - 
Method in class bioroot. SearchBean 
  
   getSearchHash()   - 
Static method in class bioroot.antibody. AntibodyBase 
  
   getSearchHash()   - 
Static method in class bioroot.oligo. OligoBase 
  
   getSearchHash()   - 
Static method in class bioroot.plasmid. PlasmidBase 
  
   getSearchHash()   - 
Static method in class bioroot.strain. StrainBase 
  
   getSearchNames()   - 
Static method in class bioroot.antibody. AntibodyBase 
  
   getSearchNames()   - 
Static method in class bioroot.oligo. OligoBase 
  
   getSearchNames()   - 
Static method in class bioroot.plasmid. PlasmidBase 
  
   getSearchNames()   - 
Static method in class bioroot.strain. StrainBase 
  
   getSeedType()   - 
Method in class bioroot.strain. StrainBean 
  
   getSegmentIndexes()   - 
Method in class util.bio.wrappers. Segment 
  
   getSelexOligos()   - 
Method in class util.bio.xamer. Xamer 
  
   getSeq()   - 
Method in class meme. MotifHit 
  
   getSeq()   - 
Method in class selex. SubSeq 
  
   getSeq()   - 
Method in class util.bio.parsers. Fasta 
  
   getSeq1()   - 
Method in class gata.aligner. Alignment 
  
   getSeq1()   - 
Method in class util.bio.wrappers. BL2SeqHit 
  
   getSeq2()   - 
Method in class gata.aligner. Alignment 
  
   getSeq2()   - 
Method in class util.bio.wrappers. BL2SeqHit 
  
   getSeqFile(int)   - 
Method in class selex. SelexParams 
  
   getSeqFile()   - 
Method in class selex. SeqFiles 
  
   getSeqFiles()   - 
Method in class selex. SelexParams 
  
   getSeqId()   - 
Method in class util.bio.parsers.gff. Gff3Feature 
  
   getSeqName()   - 
Method in class util.bio.parsers.gff. GffFeature 
  
   getSeqRead()   - 
Method in class selex. SubSeq 
  
   getSeqReads()   - 
Method in class selex. SeqFiles 
  
   getSeqs()   - 
Method in class util.bio.parsers. MultiFastaParser 
  
   getSeqsAL()   - 
Method in class util.bio.parsers. MultiFastaParser 
  
   getSeqsNames()   - 
Method in class util.bio.parsers. MultiFastaParser 
 Returns a hashmap of the fasta sequence:name
   getSequence()   - 
Method in class bioroot. BlastMatcher 
  
   getSequence()   - 
Method in class bioroot.oligo. OligoBean 
  
   getSequence()   - 
Method in class bioroot.plasmid. PlasmidBean 
  
   getSequence()   - 
Method in class edu.utah.seq.data. EnrichedRegion 
  
   getSequence()   - 
Method in class edu.utah.seq.methylation454. BaseRead 
  
   getSequence()   - 
Method in class edu.utah.seq.methylation454. DataLine 
  
   getSequence()   - 
Method in class edu.utah.seq.methylation454. Read 
  
   getSequence()   - 
Method in class edu.utah.seq.methylation454. Reference 
  
   getSequence()   - 
Method in class trans.bkgrnd. BackGroundOligo 
  
   getSequence()   - 
Method in class trans.bkgrnd. LoadedOligo 
  
   getSequence()   - 
Method in class trans.main. Interval 
  
   getSequence()   - 
Method in class trans.main. Oligo 
  
   getSequence()   - 
Method in class trans.tpmap. TPMapFeature 
  
   getSequence()   - 
Method in class trans.tpmap. TPMapLine 
  
   getSequence()   - 
Method in class util.bio.seq. IndexedSequence 
  
   getSequence()   - 
Method in class util.bio.wrappers. Primer3Pick 
  
   getSerializedCelFile()   - 
Method in class trans.qc. CelFileStats 
  
   getSetOneFile()   - 
Method in class trans.graphics. RankedSetAnalysis 
  
   getSetTwoFile()   - 
Method in class trans.graphics. RankedSetAnalysis 
  
   getShapes()   - 
Method in class gata.aligner. Alignment 
  
   getSimpleMulti()   - 
Method in class util.amazon. S3Manager 
  
   getSize()   - 
Method in class util.apps. HouseBean 
  
   getSize()   - 
Method in class util.gen. FileSize 
  
   getSizeMappedFeatures()   - 
Method in class trans.cel. CelMapper 
  
   getSizeOfOligo()   - 
Method in class trans.main. T2Parameter 
  
   getSizeOfOligoMinusOne()   - 
Method in class trans.main. Interval 
  
   getSliders()   - 
Method in class gata.plotter. Sliders 
  
   getSlope()   - 
Method in class util.gen. LinearRegression 
  
   getSm()   - 
Method in class edu.utah.seq.data. SmoothingWindowInfo 
  
   getSmallestAlignPanelY()   - 
Method in class gata.main. GATAParams 
  
   getSmallestGlyphPanelY()   - 
Method in class gata.main. AnnoSpecParams 
  
   getSmoothedScore()   - 
Method in class trans.main. Oligo 
  
   getSortBy()   - 
Method in class bioroot. BlastMatcher 
  
   getSortBy()   - 
Method in class trans.main. Interval 
  
   getSortBy()   - 
Method in class trans.main. Window 
  
   getSortBy()   - 
Method in class util.bio.parsers.gff. Gff3Feature 
  
   getSource()   - 
Method in class bioroot.antibody. AntibodyBean 
  
   getSource()   - 
Method in class bioroot.plasmid. PlasmidBean 
  
   getSource()   - 
Method in class bioroot.strain. StrainBean 
  
   getSource()   - 
Method in class cgar.beans. GenomeBean 
  
   getSource()   - 
Method in class util.bio.parsers.gff. Gff3Feature 
  
   getSource()   - 
Method in class util.bio.parsers.gff. GffFeature 
  
   getSpace()   - 
Static method in class util.apps.gump. Option 
  
   getSpeciesId()   - 
Method in class cgar.beans. GenomeBean 
  
   getSpline()   - 
Method in class trans.cel. SplineScalar 
  
   getSQL()   - 
Method in class cgar. Util 
 Make connection to CGAR DB.
   getStart()   - 
Method in class edu.utah.seq.data. EnrichedRegion 
  
   getStart()   - 
Method in class edu.utah.seq.data. SmoothingWindow 
  
   getStart()   - 
Method in class expr. CorrelationWindow 
  
   getStart()   - 
Method in class gata.geneGlyphs. GeneRepGlyph 
  
   getStart()   - 
Method in class meme. MotifHit 
  
   getStart()   - 
Method in class trans.anno. BindingRegion 
  
   getStart()   - 
Method in class trans.anno. Region 
  
   getStart()   - 
Method in class trans.graphics. GenomicRegion 
  
   getStart()   - 
Method in class trans.main. Oligo 
  
   getStart()   - 
Method in class trans.main. PosRegion 
  
   getStart()   - 
Method in class trans.roc. Bin 
  
   getStart()   - 
Method in class trans.roc. Positive 
  
   getStart()   - 
Method in class trans.roc. RocWindow 
  
   getStart()   - 
Method in class trans.tpmap. TPMapLine 
  
   getStart()   - 
Method in class util.bio.annotation. Coordinate 
  
   getStart()   - 
Method in class util.bio.annotation. ExonIntron 
  
   getStart()   - 
Method in class util.bio.annotation. GeneGroup 
  
   getStart()   - 
Method in class util.bio.annotation. GenericFeature 
  
   getStart()   - 
Method in class util.bio.annotation. StartStop 
  
   getStart()   - 
Method in class util.bio.annotation. Transcript 
  
   getStart()   - 
Method in class util.bio.annotation. Translation 
  
   getStart()   - 
Method in class util.bio.parsers.gff. Gff3Feature 
  
   getStart()   - 
Method in class util.bio.parsers.gff. GffFeature 
  
   getStart()   - 
Method in class util.bio.seq. IndexedSequence 
  
   getStart()   - 
Method in class util.gen. Bin 
  
   getStart1stOligo()   - 
Method in class trans.main. Interval 
  
   getStart1stOligo()   - 
Method in class trans.main. Window 
  
   getSTART_INDEX_COMPSEQ()   - 
Method in class gata.aligner. AlignParams 
  
   getSTART_INDEX_REFSEQ()   - 
Method in class gata.aligner. AlignParams 
  
   getStartATGPosition()   - 
Method in class util.bio.annotation. GeneRep 
 Conservative estimate of the start ATG position, from the longest transcript
   getStartEnd()   - 
Method in class util.bio.annotation. StartStop 
  
   getStartExactMatch()   - 
Method in class trans.tpmap. BlastFilterResult 
  
   getStartIndex()   - 
Method in class trans.cel. ChromosomeIndex 
  
   getStartLastOligo()   - 
Method in class trans.main. Interval 
  
   getStartLastOligo()   - 
Method in class trans.main. Window 
  
   getStartPosition()   - 
Method in class edu.utah.seq.methylation454. Reference 
  
   getStartPosition()   - 
Method in class trans.bkgrnd. LoadedOligo 
  
   getStartSeq1()   - 
Method in class util.bio.wrappers. BL2SeqHit 
  
   getStartSeq2()   - 
Method in class util.bio.wrappers. BL2SeqHit 
  
   getStartStop()   - 
Method in class gata.plotter. StartStop 
  
   getState()   - 
Method in class util.apps. HouseBean 
  
   getStats()   - 
Method in class trans.cel. ControlStats 
  
   getStatus()   - 
Method in class util.apps. HouseBean 
  
   getStndDev()   - 
Method in class trans.qc. StatFlag 
  
   getStop()   - 
Method in class edu.utah.seq.data. EnrichedRegion 
  
   getStop()   - 
Method in class edu.utah.seq.data. SmoothingWindow 
  
   getStop()   - 
Method in class expr. CorrelationWindow 
  
   getStop()   - 
Method in class meme. MotifHit 
  
   getStop()   - 
Method in class trans.graphics. GenomicRegion 
  
   getStop()   - 
Method in class trans.roc. Bin 
  
   getStop()   - 
Method in class trans.roc. Positive 
  
   getStop()   - 
Method in class util.bio.annotation. Coordinate 
  
   getStop()   - 
Method in class util.bio.seq. IndexedSequence 
  
   getStop()   - 
Method in class util.gen. Bin 
  
   getStopIndex()   - 
Method in class trans.cel. ChromosomeIndex 
  
   getStopSeq1()   - 
Method in class util.bio.wrappers. BL2SeqHit 
  
   getStopSeq2()   - 
Method in class util.bio.wrappers. BL2SeqHit 
  
   getStorageLoc()   - 
Method in class gata.aligner. AlignerPreferences 
  
   getStrainBeans()   - 
Method in class bioroot.strain. StrainUploadBean 
  
   getStrainConcats(DBUtil)   - 
Method in class bioroot.antibody. AntibodyBean 
 Returns an array of id: strainName, or empty String[]{""} if there are none.
   getStrainConcats(DBUtil)   - 
Method in class bioroot.plasmid. PlasmidBean 
 Returns an array of id: strainName, or empty String[]{""} if there are none.
   getStrainForm(StrainBean, DBUtil, UserBean)   - 
Static method in class bioroot.strain. StrainBase 
 Returns a strain form filled with the values in the  bean.
   getStrainPreference()   - 
Method in class bioroot. UserBean 
  
   getStrainPrefs()   - 
Method in class bioroot. UserBean 
  
   getStrainReport(StrainBean, DBUtil, UserBean, boolean)   - 
Static method in class bioroot.strain. StrainBase 
 Returns an strain report.
   getStrainReports()   - 
Method in class bioroot.strain. StrainUploadBean 
  
   getStrainTypes()   - 
Method in class bioroot.strain. StrainPreference 
  
   getStrand()   - 
Method in class edu.utah.seq.data. Info 
  
   getStrand()   - 
Method in class edu.utah.seq.methylation454. BaseRead 
  
   getStrand()   - 
Method in class edu.utah.seq.methylation454. DataLine 
  
   getStrand()   - 
Method in class edu.utah.seq.methylation454. Read 
  
   getStrand()   - 
Method in class edu.utah.seq.parsers. BarParser 
  
   getStrand()   - 
Method in class trans.main. T2Parameter 
  
   getStrand()   - 
Method in class trans.roc. ControlGene 
  
   getStrand()   - 
Method in class util.bio.annotation. GenericFeature 
  
   getStrand()   - 
Method in class util.bio.parsers.gff. Gff3Feature 
  
   getStrand()   - 
Method in class util.bio.parsers.gff. GffFeature 
  
   getStrand()   - 
Method in class util.bio.parsers. UCSCGeneLine 
  
   getStrand()   - 
Method in class util.bio.wrappers. BlastResult 
  
   getStreetAddress()   - 
Method in class util.apps. HouseBean 
  
   getStrippedRecogSeq()   - 
Method in class util.bio.digest. Enzyme 
  
   getSubmitterUserBean()   - 
Method in class bioroot. UserRegistrationBean 
  
   getSubSeq()   - 
Method in class selex. SubSeq 
  
   getSubWindows()   - 
Method in class edu.utah.seq.data. EnrichedRegion 
  
   getSubWindowSize()   - 
Method in class trans.main. T2Parameter 
  
   getSymmetricNullApp()   - 
Method in class trans.main. T2Parameter 
  
   getT()   - 
Method in class util.bio.parsers. BaseCount 
  
   getTableName()   - 
Method in class bioroot. SearchBean 
  
   getTagValues()   - 
Method in class edu.utah.seq.parsers. BarParser 
  
   getTarget()   - 
Method in class util.bio.parsers.gff. Gff3Feature 
 Reserved Attribute.
   getTargetMedian()   - 
Method in class trans.cel. QuantileNormalization 
  
   getTargetMedian()   - 
Method in class trans.main. T2Parameter 
  
   getTargets()   - 
Method in class expr. MiRNA 
  
   getTextArea()   - 
Method in class gata.plotter. Console 
  
   getTextArea()   - 
Method in class util.apps.gump. GumpResults 
  
   getTextArea()   - 
Method in class util.gen. TextFrame 
  
   getTextFrame()   - 
Method in class trans.graphics. RankedSetAnalysis 
  
   getThinDNALines()   - 
Method in class gata.main. AnnoSpecParams 
  
   getThinProteinLines()   - 
Method in class gata.main. AnnoSpecParams 
  
   getThinRNALines()   - 
Method in class gata.main. AnnoSpecParams 
  
   getThinStroke()   - 
Method in class gata.main. GATAParams 
  
   getThreadedResults()   - 
Method in class util.amazon. S3Manager 
  
   getThreePrimeNonCodingRegion()   - 
Method in class util.bio.annotation. GeneRep 
 Conservative estimate returning smallest non coding region.
   getTitle()   - 
Method in class trans.graphics. IntervalDrawPanel 
  
   getTitle()   - 
Method in class util.bio.cluster. HierarchicalClustering 
  
   getTm()   - 
Method in class trans.bkgrnd. LoadedOligo 
  
   getToolsFrameRef()   - 
Method in class gata.main. GATAParams 
  
   getTopCluster()   - 
Method in class util.bio.cluster. Cluster 
  
   getTopCuts()   - 
Method in class util.bio.digest. Enzyme 
  
   getTotalBinCounts()   - 
Method in class util.gen. Histogram 
 Including num less than and num more than.
   getTotalClusters()   - 
Method in class util.bio.cluster. Cluster 
  
   getTotalDelta(double[][], String)   - 
Static method in class util.bio.calc. NucleicAcid 
 Returns the total delta given a lower case DNA sequence.
   getTotalNumberWindows()   - 
Method in class trans.roc. Positive 
  
   getTpmapFiles()   - 
Method in class trans.main. T2Parameter 
  
   getTrackConverter()   - 
Method in class gata.geneGlyphs. Annotation 
  
   getTrackedGenericGlyphs()   - 
Method in class gata.main. AnnoSpecParams 
  
   getTrackLabels()   - 
Method in class gata.main. AnnoSpecParams 
  
   getTrackLabelVis()   - 
Method in class gata.main. AnnoSpecParams 
  
   getTrackNumber()   - 
Method in class gata.main. AnnoSpecParams 
  
   getTrackNumber()   - 
Method in class util.bio.annotation. GenericFeature 
  
   getTrackStrokes()   - 
Method in class gata.main. AnnoSpecParams 
  
   getTrackThickness()   - 
Method in class gata.main. AnnoSpecParams 
  
   getTrackVis()   - 
Method in class gata.main. AnnoSpecParams 
  
   getTranscript()   - 
Method in class util.bio.annotation. TransGroup 
  
   getTransformedPValue()   - 
Method in class trans.main. WilcoxonSignedRankTest 
  
   getTransformedPValueString()   - 
Method in class trans.main. WilcoxonSignedRankTest 
  
   getTransGrps()   - 
Method in class util.bio.annotation. GeneGroup 
  
   getTranslation()   - 
Method in class util.bio.annotation. TransGroup 
  
   getTreatmentCelFiles()   - 
Method in class trans.main. T2Parameter 
  
   getTreatmentIntensities(int)   - 
Method in class trans.main. Oligo 
  
   getTreatmentIntensities()   - 
Method in class trans.main. ScanChromosomes 
  
   getTreatmentIntensities()   - 
Method in class trans.main. ScanChromosomesCNV 
  
   getTreatmentPoints()   - 
Method in class edu.utah.seq.data. EnrichedRegion 
  
   getTreatmentValues()   - 
Method in class trans.main. ScoredGene 
  
   getTruncatedNames(File[])   - 
Static method in class util.gen. IO 
 Fetches and truncates common chars, from file names, front and back, uses getName() so no path info is involved.
   getTruncatedNames(File[][])   - 
Static method in class util.gen. IO 
 Fetches and truncates common chars, from file names, front and back, uses getName() so no path info is involved.
   getTss()   - 
Method in class util.bio.parsers. UCSCGeneLine 
  
   getTValue()   - 
Method in class trans.main. WilcoxonSamplePair 
  
   getTxEnd()   - 
Method in class util.bio.parsers. UCSCGeneLine 
  
   getTxStart()   - 
Method in class util.bio.parsers. UCSCGeneLine 
  
   getType()   - 
Method in class bioroot.antibody. AntibodyBean 
  
   getType()   - 
Method in class bioroot. ContactBean 
  
   getType()   - 
Method in class bioroot. LoginBean 
  
   getType()   - 
Method in class bioroot.oligo. OligoBean 
  
   getType()   - 
Method in class bioroot.strain. StrainBean 
  
   getType()   - 
Method in class bioroot. UserBean 
  
   getType()   - 
Method in class bioroot. UserRegistrationBean 
  
   getType()   - 
Method in class trans.roc. RocWindow 
  
   getType()   - 
Method in class util.bio.annotation. GeneGroup 
  
   getType()   - 
Method in class util.bio.parsers.gff. Gff3Feature 
  
   getTypeId()   - 
Method in class cgar.beans. UserTypeBean 
  
   getUM()   - 
Method in class bioroot.oligo. OligoBean 
  
   getUMNgUl()   - 
Method in class bioroot.oligo. OligoBean 
  
   getUri()   - 
Method in class cgar.beans. GenomeBean 
  
   getUri()   - 
Method in class cgar.beans. ProcessedDataBean 
  
   getUri()   - 
Method in class cgar.beans. RawDataBean 
  
   getUseDilutionForm(UseDilution, StringBuffer, int, int, boolean)   - 
Static method in class bioroot.antibody. AntibodyBase 
 Returns a UseDilutionNotes where the names are apended with apbIndex.index ie 0.0, 0.1, 0.2,,1.0,1.1...
   getUseDilutionForms(AntibodyPrep, DBUtil, StringBuffer, int)   - 
Static method in class bioroot.antibody. AntibodyBase 
 Pumps out useDilution forms, should always have one with each Prep
   getUseDilutionReport(UseDilution, StringBuffer, int, int)   - 
Static method in class bioroot.antibody. AntibodyBase 
  
   getUseDilutionReports(AntibodyPrep, DBUtil, StringBuffer, int, boolean)   - 
Static method in class bioroot.antibody. AntibodyBase 
 Pumps out useDilution reports
   getUseDilutions(DBUtil)   - 
Method in class bioroot.antibody. AntibodyPrep 
  
   getUser(DBUtil, String)   - 
Static method in class bioroot. AccountManagement 
 fetch all items from a specific user
   getUserBean()   - 
Method in class bioroot. LoginBean 
  
   getUserComments()   - 
Method in class bioroot.antibody. AntibodyPrep 
  
   getUserId()   - 
Method in class bioroot. LoginBean 
  
   getUserId()   - 
Method in class bioroot. UserRegistrationBean 
  
   getUserId()   - 
Method in class cgar.beans. AnalysisBean 
  
   getUserId()   - 
Method in class cgar.beans. LoginBean 
  
   getUserInput()   - 
Method in class util.apps.gump. Option 
  
   getUserInputs()   - 
Method in class meme. MemeR 
 Uses JOptionPane dialog input boxes to get user inputs
   getUserInputS()   - 
Method in class util.apps.gump. Option 
  
   getUsers(DBUtil, int)   - 
Static method in class bioroot. AccountManagement 
 Returns a String[] of Users belonging to a particular labgroup.
   getUserTypeId()   - 
Method in class cgar. LabGroupUserType 
  
   getValues()   - 
Method in class edu.utah.seq.parsers. BarParser 
  
   getValues()   - 
Method in class expr. ExpressedGene 
  
   getValues()   - 
Method in class trans.misc. GrGraph 
  
   getValues()   - 
Method in class util.bio.cluster. Cluster 
  
   getVersionedGenome()   - 
Method in class edu.utah.seq.data. Info 
  
   getVersionedGenome()   - 
Method in class edu.utah.seq.parsers. BarParser 
  
   getViewLine(ReagentBean[], int[], Preference, String)   - 
Static method in class bioroot. Util 
 Returns hot linked html for 1-30 31-60 ...
   getViewPort()   - 
Method in class gata.geneGlyphs. GlyphPanel 
  
   getViewPort()   - 
Method in class gata.plotter. AlignPanel 
  
   getVirtualCel()   - 
Method in class trans.qc. CelFileStats 
  
   getVisableAlignments()   - 
Method in class gata.plotter. AlignPanel 
  
   getVisibilities(DBUtil, UserBean)   - 
Method in class bioroot. Preference 
  
   getVisibilities(UserBean, DBUtil)   - 
Static method in class bioroot. Util 
 If user is part of an organization, it returns WWW, orgName, Lab Mates, Private.
   getVisibility()   - 
Method in class bioroot. Preference 
  
   getVisibility(DBUtil)   - 
Method in class bioroot. ReagentBean 
  
   getVisibilityId()   - 
Method in class cgar.beans. AnalysisBean 
  
   getVolume()   - 
Method in class bioroot.oligo. OligoBean 
  
   getVolume()   - 
Method in class bioroot.plasmid. PlasmidBean 
  
   getW()   - 
Method in class trans.main. WilcoxonSignedRankTest 
  
   getW()   - 
Method in class util.bio.wrappers. BL2Seq 
  
   getWidth()   - 
Method in class gata.main. GATAParams 
  
   getWidth()   - 
Method in class util.apps.gump. GumpDrawFrame 
  
   getWidthBuffImage()   - 
Method in class gata.plotter. AlignPanel 
  
   getWIN_SIZE()   - 
Method in class gata.aligner. AlignParams 
  
   getWindow()   - 
Method in class gata.aligner. AlignerPreferences 
  
   getWindows()   - 
Method in class trans.main. ScanChromosomes 
  
   getWindows()   - 
Method in class trans.main. ScanChromosomesCNV 
  
   getWindowSize()   - 
Method in class trans.main. T2Parameter 
  
   getWMinus()   - 
Method in class trans.main. WilcoxonSignedRankTest 
  
   getWOffSet()   - 
Method in class trans.graphics. Graph 
  
   getWord()   - 
Method in class bioroot. SearchHit 
  
   getWordBoundaryMatches()   - 
Method in class bioroot. SearchHit 
  
   getWPlus()   - 
Method in class trans.main. WilcoxonSignedRankTest 
  
   getWsps()   - 
Method in class trans.main. WilcoxonSignedRankTest 
  
   getX()   - 
Method in class trans.graphics. Graph 
  
   getX()   - 
Method in class util.gen. LinearRegression 
  
   getXamer()   - 
Method in class util.bio.xamer. Xamer 
  
   getYearBuilt()   - 
Method in class util.apps. HouseBean 
  
   getYTop()   - 
Method in class gata.geneGlyphs. TransGrpGlyph 
 Top most y coordinate for this transGroup, including transGrp spacer, start
 next Shape here for no overlap
   getZipCode()   - 
Method in class util.apps. HouseBean 
  
   getZoomedWidth()   - 
Method in class gata.main. GATAParams 
  
   getZw()   - 
Method in class trans.main. WilcoxonSignedRankTest 
  
   gff(Oligo, Interval, String, int, StringBuffer)   - 
Static method in class trans.main. IntervalGFFPrinter 
 Appends a GFF3 line for an Oligo.
   gff(BindingPeak, Interval, String, int, StringBuffer)   - 
Static method in class trans.main. IntervalGFFPrinter 
 Appends a GFF3 line for a BindingPeak
   gff(SubWindow, Interval, String, StringBuffer)   - 
Static method in class trans.main. IntervalGFFPrinter 
 Appends a GFF3 line for a SubWindow.
   gff(Interval, int, String, StringBuffer)   - 
Method in class trans.main. IntervalGFFPrinter 
 Appends a GFF3 line for an Interval.
   gff(Interval, String, StringBuffer)   - 
Method in class trans.main. IntervalGFFPrinter 
 Appends a GFF3 line for a Window.
   Gff2ToBindingRegions   - Class in  trans.anno  Converts a gff2 file to a binding region file (chr start end mockSequence).   Gff2ToBindingRegions()   - 
Constructor for class trans.anno. Gff2ToBindingRegions 
  
   Gff2ToIntervals   - Class in  trans.anno  Converts a gff2 file to an interval object file.   Gff2ToIntervals()   - 
Constructor for class trans.anno. Gff2ToIntervals 
  
   Gff3Feature   - Class in  util.bio.parsers.gff  A container for each of the values in a GFF3 line.   Gff3Feature()   - 
Constructor for class util.bio.parsers.gff. Gff3Feature 
  
   Gff3Feature(String)   - 
Constructor for class util.bio.parsers.gff. Gff3Feature 
 Always check if the GFF3Feature is valid after instantiating with this constructor.
   Gff3Parser   - Class in  util.bio.parsers.gff  For parsing a GFF3 text file into GFF3Feature objects, be sure to set the regExTypes String filter to match which types you want to save!   Gff3Parser(File)   - 
Constructor for class util.bio.parsers.gff. Gff3Parser 
  
   Gff3Parser()   - 
Constructor for class util.bio.parsers.gff. Gff3Parser 
  
   GffFeature   - Class in  util.bio.parsers.gff  A container for each of the values in a GFF 2 line.   GffFeature(String)   - 
Constructor for class util.bio.parsers.gff. GffFeature 
  
   GffParser   - Class in  util.bio.parsers.gff  Parses GFF files extracting the annotation according to GFF(2).   GffParser(File, int, int)   - 
Constructor for class util.bio.parsers.gff. GffParser 
  
   GlyphPanel   - Class in  gata.geneGlyphs      GlyphPanel(AnnoSpecParams, GATAParams)   - 
Constructor for class gata.geneGlyphs. GlyphPanel 
  
   Gr   - Class in  trans.roc  Gr line container.   Gr(String)   - 
Constructor for class trans.roc. Gr 
  
   Gr(int, float)   - 
Constructor for class trans.roc. Gr 
  
   Gr2Bar   - Class in  trans.misc      Gr2Bar(String[])   - 
Constructor for class trans.misc. Gr2Bar 
  
   Graph   - Class in  trans.graphics  Graph for   IntervalPlotter  .   Graph(double[], double[], double, double, Color)   - 
Constructor for class trans.graphics. Graph 
  
   GRAPH_MAX_Y_TAG   - 
Static variable in class edu.utah.seq.parsers. BarParser 
  
   GRAPH_MIN_Y_TAG   - 
Static variable in class edu.utah.seq.parsers. BarParser 
  
   GRAPH_TYPE_BAR   - 
Static variable in class edu.utah.seq.parsers. BarParser 
  
   GRAPH_TYPE_COLOR_TAG   - 
Static variable in class edu.utah.seq.parsers. BarParser 
  
   GRAPH_TYPE_DOT   - 
Static variable in class edu.utah.seq.parsers. BarParser 
  
   GRAPH_TYPE_HEATMAP   - 
Static variable in class edu.utah.seq.parsers. BarParser 
  
   GRAPH_TYPE_LINE   - 
Static variable in class edu.utah.seq.parsers. BarParser 
  
   GRAPH_TYPE_MINMAXAVE   - 
Static variable in class edu.utah.seq.parsers. BarParser 
  
   GRAPH_TYPE_STAIRSTEP   - 
Static variable in class edu.utah.seq.parsers. BarParser 
  
   GRAPH_TYPE_TAG   - 
Static variable in class edu.utah.seq.parsers. BarParser 
  
   GrComparator   - Class in  trans.roc      GrComparator()   - 
Constructor for class trans.roc. GrComparator 
  
   GrGraph   - Class in  trans.misc  Container for holding info related to a xxx.gr file.   GrGraph(String, String, int[], float[])   - 
Constructor for class trans.misc. GrGraph 
  
   GrGraph(String, String, Gr[])   - 
Constructor for class trans.misc. GrGraph 
  
   groupName(RandomizeLabels.ArrayName[])   - 
Method in class trans.main. RandomizeLabels 
 Returns a sorted groupName where the sort is on the treatments and controls, not both.
   growIntArrays(int[][])   - 
Static method in class gata.plotter. ConservedSeqs 
  
   growLeft(int)   - 
Method in class trans.main. PeakPicker 
 Uses the idea of a local window, counts the number of pos slopes within the window, if > minPercent, keeps advancing.
   growRight(int)   - 
Method in class trans.main. PeakPicker 
 Uses the idea of a local window, counts the number of neg slopes within the window, if > minPercent, keeps advancing.
   Gump   - Class in  util.apps.gump      Gump(String[])   - 
Constructor for class util.apps.gump. Gump 
  
   GumpApplicationPanel   - Class in  util.apps.gump      GumpApplicationPanel(Gump, GumpDrawFrame)   - 
Constructor for class util.apps.gump. GumpApplicationPanel 
  
   GumpDrawFrame   - Class in  util.apps.gump  Frame for  RankedSetAnalysis .   GumpDrawFrame(Gump)   - 
Constructor for class util.apps.gump. GumpDrawFrame 
  
   GumpResults   - Class in  util.apps.gump      GumpResults(int, int, int, int, String)   - 
Constructor for class util.apps.gump. GumpResults 
  
   GumpResultsScrollPane   - Class in  util.apps.gump      GumpResultsScrollPane(Gump)   - 
Constructor for class util.apps.gump. GumpResultsScrollPane 
  
   GumpToolPanel   - Class in  util.apps.gump      GumpToolPanel()   - 
Constructor for class util.apps.gump. GumpToolPanel 
  
 
 


 
   
  
 
 
 
   
 
   
          Overview     
        Package   
        Class   
        Use   
          Tree     
          Deprecated     
       Index    
          Help     
   
 
 
  
 
 
 

 
  
   PREV LETTER   
   NEXT LETTER    
  
    FRAMES     
   NO FRAMES     
  
    All Classes  ');
  }
  //-->
 
 
    All Classes  
 


  
 
 
  
 

 A   B   C   D   E   F   G   H   I   J   L   M   N   O   P   Q   R   S   T   U   V   W   X   Z
